# Identification of Catechins’ Binding Sites in Monomeric A*β*_42_ through Ensemble Docking and MD Simulations

**DOI:** 10.3390/ijms24098161

**Published:** 2023-05-03

**Authors:** Rohoullah Firouzi, Shahin Sowlati-Hashjin, Cecilia Chávez-García, Mitra Ashouri, Mohammad Hossein Karimi-Jafari, Mikko Karttunen

**Affiliations:** 1 Department of Physical Chemistry, Chemistry and Chemical Engineering Research Center of Iran, Tehran 1496813151, Iran; 2 Institute of Biomedical Engineering, University of Toronto, Toronto, ON M5S 3G9, Canada; shahin.sowlati@utoronto.ca; 3 Department of Chemistry, The University of Western Ontario, 1151 Richmond Street, London, ON N6A 5B7, Canada; cecilia.uku@gmail.com; 4 The Centre of Advanced Materials and Biomaterials Research, The University of Western Ontario, 1151 Richmond Street, London, ON N6A 5B7, Canada; 5 Department of Physical Chemistry, School of Chemistry, College of Science, University of Tehran, Tehran P.O. Box 14155-6619, Iran; m.ashouri@ut.ac.ir; 6 Department of Bioinformatics, Institute of Biochemistry and Biophysics, University of Tehran, Tehran P.O. Box 14155-6619, Iran; mhkarimijafari@ut.ac.ir; 7 Department of Physics and Astronomy, The University of Western Ontario, 1151 Richmond Street, London, ON N6A 3K7, Canada

**Keywords:** catechins, amyloid-*β*, binding sites, ensemble docking, MD simulations

## Abstract

The assembly of the amyloid-*β* peptide (A*β*) into toxic oligomers and fibrils is associated with Alzheimer’s disease and dementia. Therefore, disrupting amyloid assembly by direct targeting of the A*β* monomeric form with small molecules or antibodies is a promising therapeutic strategy. However, given the dynamic nature of A*β*, standard computational tools cannot be easily applied for high-throughput structure-based virtual screening in drug discovery projects. In the current study, we propose a computational pipeline—in the framework of the ensemble docking strategy—to identify catechins’ binding sites in monomeric A*β*_42_. It is shown that both hydrophobic aromatic interactions and hydrogen bonding are crucial for the binding of catechins to A*β*_42_. Additionally, it has been found that all the studied ligands, especially *EGCG*, can act as potent inhibitors against amyloid aggregation by blocking the central hydrophobic region of A*β*. Our findings are evaluated and confirmed with multi-microsecond MD simulations. Finally, it is suggested that our proposed pipeline, with low computational cost in comparison with MD simulations, is a suitable approach for the virtual screening of ligand libraries against A*β*.

## 1. Introduction

Intrinsically disordered proteins (IDPs) are very flexible biomolecules without a well-defined folded structure and typically have important roles in biological processes, in particular in cellular signaling and gene regulation [1,2,3,4]. Under certain conditions, some IDPs may aggregate into highly toxic oligomers. These oligomers are associated with a wide range of serious human diseases such as cancer, neurodegenerative diseases, autoimmune disorders, cardiovascular disease, and type II diabetes [4,5,6,7,8,9]. Thus, preventing or reducing aggregation of the IDPs involved in such diseases is as an effective therapeutic strategy.

In recent years, there have been efforts to design and synthesize small molecules and short peptides to block IDP aggregation at different stages along the aggregation pathway, in particular nucleation and oligomer formation [4,10,11,12,13,14,15,16,17]. Several studies have revealed that a large number of natural compounds derived from plants, animals and microorganisms have the potential to inhibit oligomerization [11,12,18,19,20]. For example, several computational and experimental observations have shown that polyphenolic plant compounds which occur naturally in fruit, vegetables, chocolate, and tea are capable of inhibiting IDP aggregation [12,13,20,21,22,23,24,25,26,27,28,29,30,31,32,33,34,35,36,37,38].

Finding aggregation inhibitors and direct targeting of monomeric IDPs via small molecules is a very active area of research and a wide variety of computational techniques have been applied, yet there are many inherent difficulties [12,13,14,27,39,40,41,42,43,44,45,46]. For example, most docking algorithms employ the flexible ligand and rigid receptor paradigm [47,48,49]. However, IDPs display high conformational heterogeneity, and ligand binding causes large structural changes in the IDP conformations. To circumvent this problem, heterogeneous conformational ensembles of IDPs have been used for docking studies [27,38,42,50,51]. Nevertheless, challenges associated with generating and choosing a set of suitable conformations for docking still remain. It will also be interesting to see how and if Alphafold [52] will change the situation as it has already been applied to non-IDP-related binding problems [53], but IDPs appear to be a challenge even for Alphafold [54].

Several methods have been proposed for efficient sampling of IDP’s conformational space and constructing a representative conformational ensemble. Examples include replica-exchange- [55,56,57,58] and metadynamics-based methods [59,60,61], diffusion map approaches [62,63] and Markov state modeling [64,65,66,67]. For comparisons between the methods, see for example [43,68,69,70]. One alternative for effective exploration of the conformational space is the use of multiple conventional MD trajectories (replicas) with different initial conditions (different velocities or/and different starting configurations). The strategy of choosing the initial conditions controls the effectiveness of this approach and its ability to enhance conformational sampling performance [71,72,73,74,75,76]. We have recently proposed a new efficient algorithm for comprehensive exploring of the conformational space of IDPs, called Blockwise Excursion Sampling (BES) [75,76]. It uses simulated annealing (SA) to find different low-energy states of various regions of conformational space as optimal starting configurations for short conventional MD simulations. In BES, conformational sampling is based on many uncorrelated short MD simulations starting from different points of the accessible phase space. It has been shown that the protocol is successful in generating a diverse conformational library for IDP conformations in agreement with experimental data [75,76].

In this work, a computational pipeline in the framework of the ensemble docking strategy has been proposed to identify catechins’ binding sites on the full-length human amyloid-*β* (A*β*_42_) monomer which is involved in Alzheimer’s disease [2,5,11,12,27,39,41,43]. Catechins (or flavan-3-ols) are dietary polyphenolic compounds commonly found in green tea and their inhibitory effects on A*β* aggregation have been the subject of numerous experimental and computational investigations [21,28,36,77,78,79,80,81,82,83,84,85,86,87,88,89,90,91,92,93,94]. There is experimental evidence indicating that the catechins are able to disturb A*β* aggregation and change the aggregation process toward the formation of non-toxic oligomers [28,37,77,78,80,81,85,86]. Several computational studies have proposed that green tea catechin EGCG interacts with A*β*_42_ through both multiple hydrophobic interactions and hydrogen bonding [28,79,84,85]. Those studies have suggested that π-stacking interactions with aromatic amino acids of the aromatic hydrophobic core region of A*β*_42_ (from Tyr10 to Phe20) affect A*β*_42_ aggregation and can, consequently, disrupt interchain interactions in A*β*_42_ protofibril structure and lead to the distortion of the protofibril structure [28,83,84,85,87,93]. Here, we applied the BES protocol to generate a reliable structural library for A*β*_42_ monomer. Through the ensemble docking approach, a catechin library was docked onto the surfaces of a library of A*β*_42_ monomers to identify the binding “hot spots” on the A*β*_42_ peptide. In order to further evaluate the docking results and the stability of complexes, the obtained structures with the largest binding energies for each A*β*_42_–catechin complex were used as the starting structures for long multi-microsecond MD simulations (total of 15 μs).

## 2. Results and Discussion

### 2.1. Docking Analysis

Structural Analysis and Identifying Ligand Binding Site

Determination of the important residues that are in close contact with the ligand is very important for the identification of potential inhibitors of A*β*_42_ aggregation. The distances between the heavy atoms of the ligand and the residues of A*β*_42_ were used to define the binding sites of A*β*_42_. Based on this assumption, a list of binding residues for each selected complex was generated for which at least one heavy atom of the residues falls within the distance cutoff of any ligand heavy atom. To assess the effect of the distance cutoff, five distances were evaluated: 3.0, 3.5, 4.0, 4.5, and 5.0 Å; the distances cutoff from 3.0 to 5.0 Å are commonly used to study ligand–protein binding interactions, such as hydrogen bonds, hydrophobic contacts, and aromatic interactions [22,95,96,97,98,99,100].

The number of contacts between each ligand and A*β*_42_ residues for the set-1 (as defined in Section 3.2) was counted for five different distance cutoffs and tabulated in Tables S1–S5. Here, we only show the results related to the distance cutoff of 5.0 Å in Table 1. It is very important to emphasize that in our structural analysis, none of the conformers selected from molecular docking alone were used to draw qualitative conclusions about the binding site. All the structural analyses in this study are based on the results given in Table 1, which were obtained from the statistical study of all the selected complexes based on ΔΔ*G_binding_*, which is illustrated in more detail in Section 3.2.

**Figure 1:**
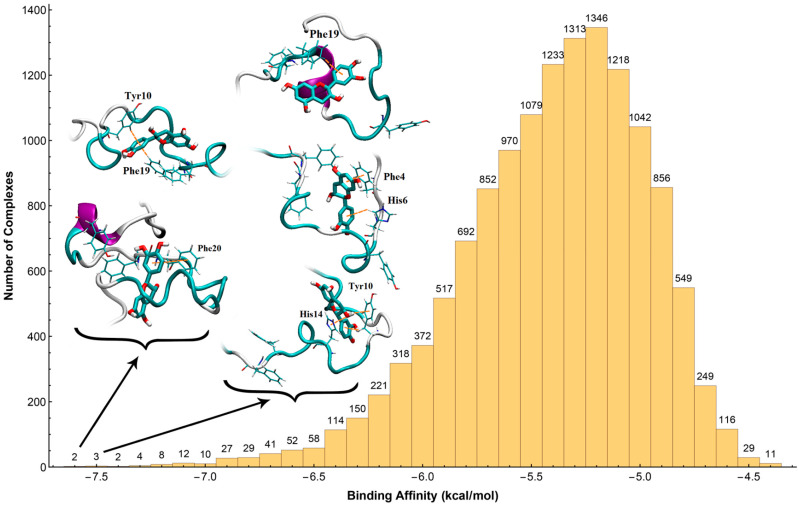
The histogram of binding affinity (kcal.mol^−1^) of the *C* ligand in set-1 (with the cutoff value of 0.1 kcal.mol^−1^, as illustrated in Section 3.2). The binding affinities were divided to bins of 0.1 kcal/mol, and the numbers on top of the bins show the number of complexes in each bin. The three aromatic residue hotspots (i.e., Tyr10, Phe19, and Phe20, see Figure 7 for sequence) and interacting aromatic residues with the ligand for some complexes with the highest binding affinities are shown in licorice representation. Favorable aromatic interactions between the ligand and peptide are depicted with orange dashed lines.

**Figure 2:**
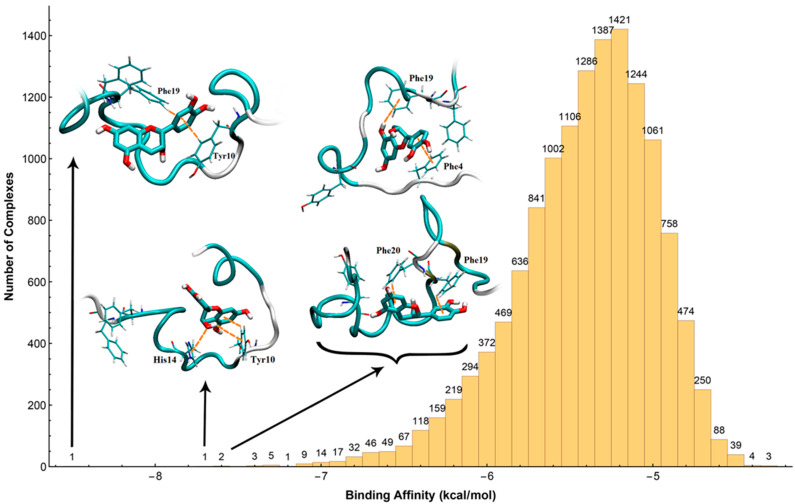
The histogram of binding affinity (kcal.mol^−1^) of the *EC* ligand in set-1 (with the cutoff value of 0.1 kcal.mol^−1^, as illustrated in Section 3.2). The binding affinities were divided to bins of 0.1 kcal.mol^−1^, and the numbers on top of the bins show the number of complexes in each bin. The three aromatic residue hotspots (i.e., Tyr10, Phe19, and Phe20**,** see Figure 7 for sequence) and interacting aromatic residues with the ligand for some complexes with the highest binding affinities are shown in licorice representation. Favorable aromatic interactions between the ligand and peptide are depicted with orange dashed lines.

**Figure 3:**
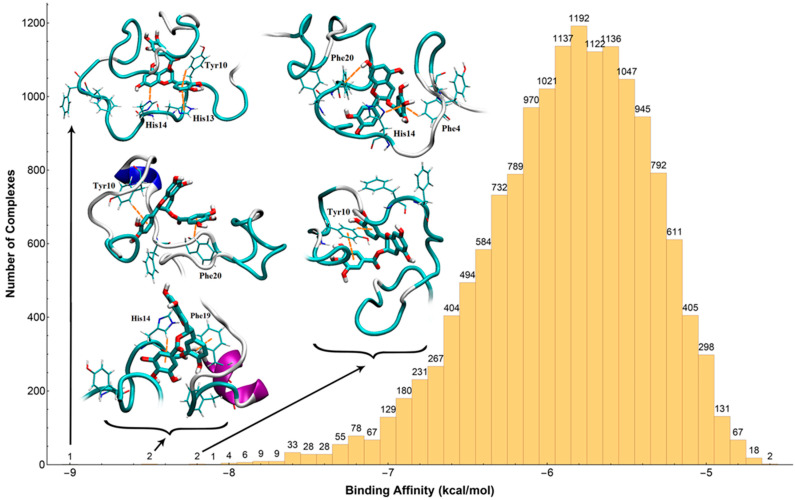
The histogram of binding affinity (kcal.mol^−1^) of the *ECG* ligand in set-1 (with the cutoff value of 0.1 kcal.mol^−1^, as illustrated in Section 3.2). The binding affinities were divided to bins of 0.1 kcal.mol^−1^, and the numbers on top of the bins show the number of complexes in each bin. The three aromatic residue hotspots (i.e., Tyr10, Phe19, and Phe20, see Figure 7 for sequence) and interacting aromatic residues with the ligand for some complexes with the highest binding affinities are shown in licorice representation. Favorable aromatic interactions between the ligand and peptide are depicted with orange dashed lines.

**Figure 4:**
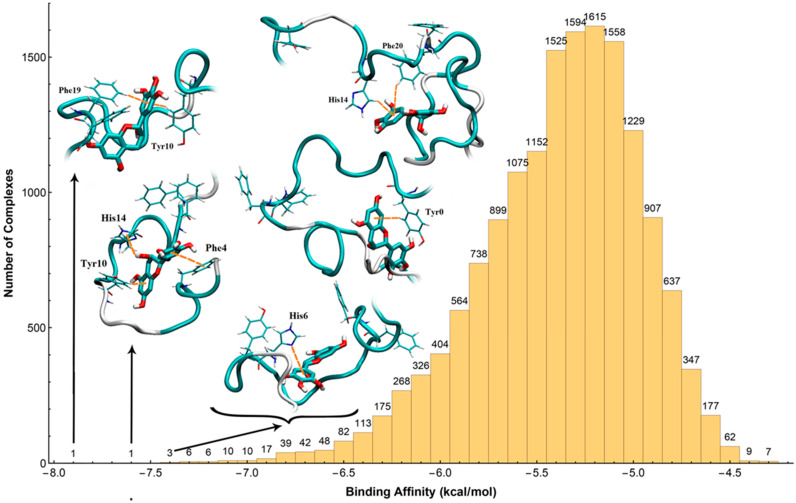
The histogram of binding affinity (kcal.mol^−1^) of the *EGC* ligand in set-1 (with the cutoff value of 0.1 kcal.mol^−1^, as illustrated in Section 3.2). The binding affinities were divided to bins of 0.1 kcal.mol^−1^, and the numbers on top of the bins show the number of complexes in each bin. The three aromatic residue hotspots (i.e., Tyr10, Phe19, and Phe20, see Figure 7 for sequence) and interacting aromatic residues with the ligand for some complexes with the highest binding affinities are shown in licorice representation. Favorable aromatic interactions between the ligand and peptide are depicted with orange dashed lines.

**Figure 5:**
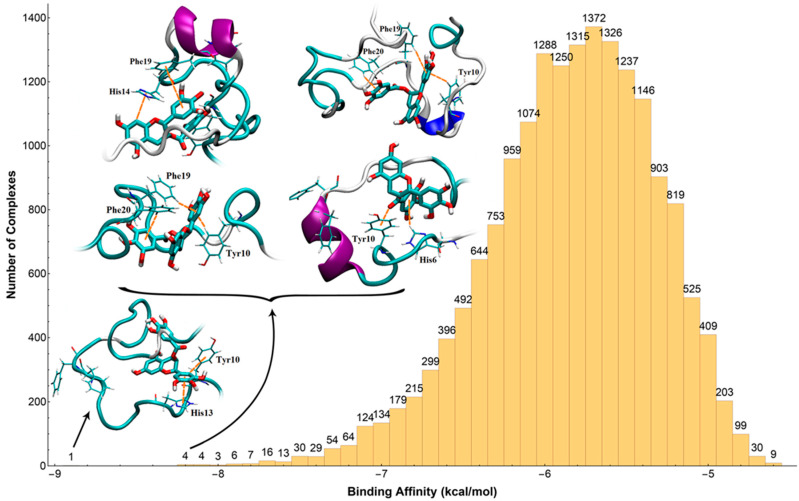
The histogram of binding affinity (kcal.mol^−1^) of the *EGCG* ligand in set-1 (with the cutoff value of 0.1 kcal.mol^−1^, as illustrated in Section 3.2). The binding affinities were divided to bins of 0.1 kcal.mol^−1^, and the numbers on top of the bins show the number of complexes for each bin. The three aromatic residue hotspots (i.e., Tyr10, Phe19, and Phe20, see Figure 7 for sequence) and interacting aromatic residues with the ligand for some complexes with the highest binding affinities are shown in licorice representation. Favorable aromatic interactions between the ligand and peptide are depicted with orange dashed lines.

The first observation from the tables is that all the ligands have the most contacts with residues Tyr10, Phe19, and Phe20. Moreover, based on the ranked lists (Table 11 and Tables S1–S5), other aromatic residues (His13, His14, His6, and Phe4) show a relatively high number of contacts and thus, they contribute to stabilizing the interactions with catechins. Therefore, it seems that these polyphenolic compounds tend to interact with the aromatic residues through stacking and/or T-shaped interactions, as shown in the snapshots of Figure 1, Figure 2, Figure 3, Figure 4 and Figure 5. Another important observation is that the tendency of ligands to interact with Tyr10 correlates with the number of hydroxyl groups on the ligands. This is seen in the data in the tables: *EGCG* possesses the largest number of hydroxyl groups (8 OH’s) and has a greater tendency to interact with Tyr10 than with Phe19 or Phe20, i.e., the number of contacts between *EGCG* and Tyr10 is larger than those between *EGCG* and Phe19 or Phe20. Thus, the data appear to imply that, hydrophobic aromatic interactions and hydrogen bonding are both crucial for the binding process. Finally, a comparative look at the tabulated values immediately shows that all ligands have a tendency to associate with the hydrophobic region of A*β*_42_ spanning residues from Tyr10 to Phe20. This region contains most of the aromatic residues found in full-length A*β*. This region also encompasses the central hydrophobic region (^16^KLVFF^20^) that, based on many experimental and computational studies, is involved in the initiation of amyloid aggregation [11,12,13,22,40,101,102,103,104,105,106]. Therefore, our docking results show that all studied ligands, especially *EGCG*, can act as potent inhibitors against amyloid aggregation through blocking the central hydrophobic region. These findings are in agreement with experimental studies [28,37,78,79,80,83].

The distributions of binding affinities (kcal.mol^−1^) of all ligands in set-1 (with the cutoff value of 0.1 kcal.mol^−1^) are shown in Figure 1, Figure 2, Figure 3, Figure 4 and Figure 5. In addition, the figures show a couple of complexes with the highest binding affinities and for each complex the different types of aromatic interactions, such as π–π, XH–π (X = C, N, O), and lone pair–π interactions between the aromatic rings of the ligands, and the sidechain of aromatic residues have been highlighted by colored dashed lines for clarity. In all cases, at least one aromatic residue was in contact with the ligand and in most cases, catechin compounds possessing multiple aromatic rings were capable of interacting with several aromatic residues simultaneously. A comparative look at Figure 1, Figure 2, Figure 3, Figure 4 and Figure 5 shows that one or two rotatable aromatic rings of the ligands (phenyl ring containing R3 substitution and gallate moiety, as illustrated in Section 3.1) are essential to make the aromatic interactions between the ligands and the aromatic residues. It is reasonable to assume that these flexible rings can be adjusted during the docking process, to maximize the aromatic interaction formation. In conclusion, it seems that these aromatic interactions play an important role in binding to aggregation-prone regions of A*β*_42_ and are essential for high affinity and binding specificity.

### 2.2. Analysis of MD Simulations

In order to evaluate the docking results, microsecond-scale MD simulations were performed on A*β*_42_-L (L = *C*, *EC*, *ECG*, *EGC*, *EGCG*) structures for which the largest binding energies were obtained by the docking procedure. The initial and final MD structures are provided in Figure S1 which show that the ligands maintain their interactions with A*β*_42_ throughout the simulation. Root mean square deviation (RMSD) of A*β*_42_ indicates that in all cases the system has reached steady state (Table S6 and Figure S2). The stability of the A*β*_42_-L complexes is also reflected in the relatively low radius of gyration (~1.0 nm) of A*β*_42_ (Table S6 and Figure S3). Moreover, the steady solvent accessible surface area (SASA; with <7% fluctuations) observed for the chains provides further support for the stability and compactness of the system (Table S6 and Figure S4).

The docking results suggest that Tyr10, Phe19, and to some extent Phe20, are the main residues involved in ligand binding. Average distances between these residues and the five ligands from the MD simulations are provided in Table 2. In agreement with the results from our docking procedure, with the exception of *C* for which the distance is ~1.0 nm, the average distance between the ligands and all three residues is ~0.5 nm (Table 2 and Figures S5–S9). The time evolution of these distances and the end-to-end distance for A*β*_42_ are shown in Figures S5–S9.

Inspection of hydrogen bonds between the ligands and A*β*_42_ reveals that hydrogen bonding plays a more important role in binding of *EGCG* and *EGC* than with the other three ligands (Table 3). Binding of *C* seems to have the least dependence on the hydrogen bonding among the ligands and relies on the π–π, XH–π (X = C, O) interactions. Average number of A*β*_42_∙∙∙ligand, A*β*_42_∙∙∙solvent, and ligand∙∙∙solvent hydrogen bonds are collected in Table 3.

Figures S10–S14 depict the contributions of A*β*_42_ residues that involve hydrogen bonding with the ligands. A comparison among the five A*β*_42_∙∙∙ligand systems reveals that there are a few residues in the A*β*_42_ sequence that frequently form hydrogen bonds with the five different catechins (e.g., Glu22, Asp23, and Ala42). These intransient hydrogen bonds alternate between the ligand and the residues, which may suggest that hydrogen bonds have a less important role in the binding of the ligands to A*β*_42_ compared to stacking interactions. The average number of hydrogen bonds between all the amino acids of the peptide and ligands are depicted in Figure S15. Similar conclusion has been previously made for A*β*_42_ protofibrils and *EGCG*, where *EGCG* was shown to bind to A*β*_42_ monomer through hydrophobic, π–π stacking, and hydrogen bonds [79,83,84,85,87,107]. Moreover, same study identifies Asp1, Glu22, and Ala42 residues to form the most hydrogen bonds with *EGCG*, which except for Asp1, agrees with our observation (Figure S14). It should be noted, however, that while we have examined disordered structures in this study, Li et al. [107] considered fibrils and 25 *EGCG* molecules.

**Figure 6:**
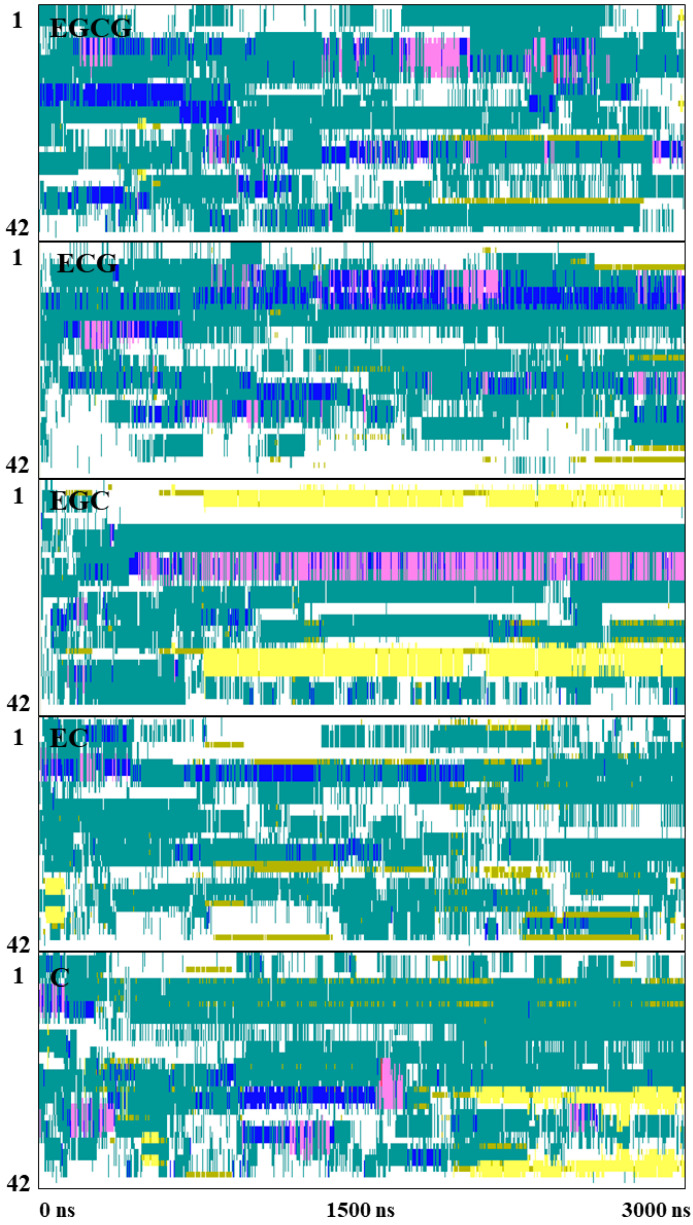
Secondary structure evolution of A*β*_42_ with time is shown in the presence of catechins. Turn, *β*-strand, *α*-helix, 3_10_ helix, random coil, and isolated bridge are shown in teal, yellow, blue, pink, white, and beige, respectively.

Finally, the secondary structure of A*β*_42_ in the presence of catechins was also evaluated and plotted using the VMD visualization software (Figure 6). As is evident from the figure, ‘turn’ and ‘coil’ are the main observed secondary structures, followed by the helices and *β*-strands. In some instances, relatively short-lived fast-alternating helices (*α*-helix and 3_10_ helix) as well as *β*-strands are formed. The propensity of certain regions of A*β*_42_ toward a specific type of secondary structure varies with the ligand type. For example, the N-terminal domain is mainly unstructured (turn and coil) in the presence of *ECG*, while a persistent *β*-strand appears in the region when *EGC* is bound. Similarly, residues 14–18 form a *β*-strand or turn-coil in the presence of *EGCG* but mainly form short-lived *α*-helices. As is apparent from the figure, different catechins have different impacts on the secondary structure of A*β*_42_, nevertheless, they all seem to impose local secondary structures across the A*β*_42_ chain, potentially impacting the formation and stability of amyloid fibrils.

## 3. Methods

### 3.1. Protein and Ligand Library Preparation

**Figure 7:**
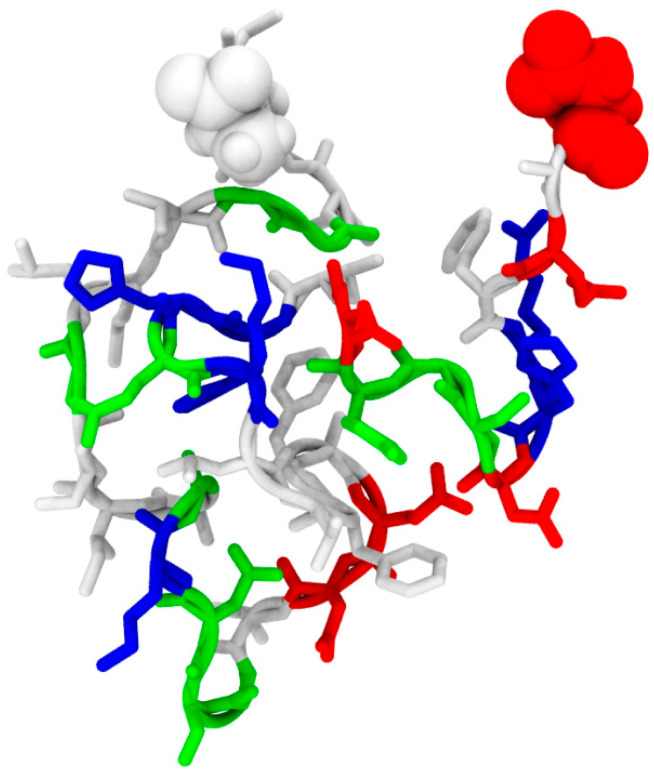
A random structure of Amyloid *β_42_*. A*β_42_* sequence: ^1^DAEFRHDSG ^10^YEVHHQKLVF ^20^FAEDVGSNKG ^30^AIIGLMVGGV ^40^VI^42^A. Asp1 (N-terminus) and Ala42 (C-terminus) are shown using van der Waals radii. Other residues are represented in licorice. Acidic, basic, polar, and non-polar amino acids are shown in red, blue, green, and white, respectively.

A library of A*β*_42_ (see Figure 7 for a snapshot and the amino acid sequence) structures was generated using the BES protocol. Briefly, 2000 excursion chains were performed such that all excursion chains were started from a fully extended structure; excursion chain refers to a sequence of MD and SA blocks in the BES protocol, for details and terminology please see [75,76]. It is interesting to note that it has already been shown that the chemical shift calculated for the conformational ensemble has a good agreement with experimental data [75,76].

**Scheme 1:**
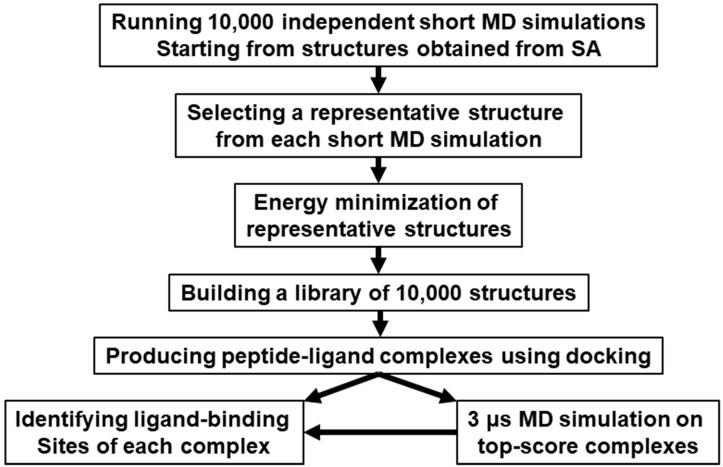
Flowchart of steps taken in this study.

Each excursion chain included five successive SA and MD blocks with maximum temperatures of 700, 600, 500, 400, and 350 K for the SA blocks. The relaxation time for each MD block was set to 120 ps and the last 100 ps were used to generate representative structures. In the next step, an average (mean) structure over the MD trajectory was obtained, and the root mean deviation (RMSD) was used as a criterion to identify the configuration in the MD trajectory that is structurally closest to the average structure. The selected structure was then energy minimized using the conjugate gradient method and used as a representative structure. As a result, for each MD block (five blocks in each excursion chain) one representative structure was derived. The final structural library included a total of 10,000 representative structures. Scheme 1 summarizes the procedure. For more technical details and a complete description of the BES protocol see [75,76].

**Figure 8:**
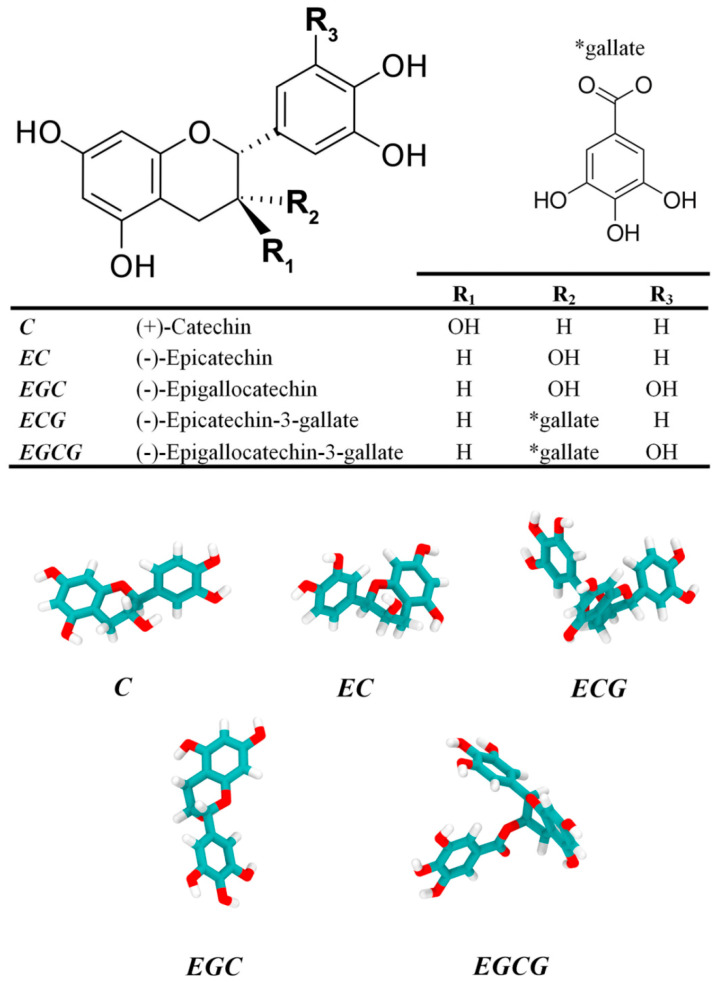
The 2D and 3D representations of catechins considered in this study.

For this study, we selected five main catechins found in green tea: (1) (+)-catechin (*C*), (2) (-)-epicatechin (*EC*), (3) (-)-epigallocatechin (*EGC*), (4) (-)-epicatechin-3-gallate (*ECG*), and (5) (-)-epigallocatechin-3-gallate (*EGCG*). Their chemical structures are shown in Figure 8 and their initial structures were taken from the ZINC database [108]. This was followed by optimization of the molecular geometries for all of them using the B3LYP exchange and correlation functional [109] and the 6-31+G(d,p) basis set. The GAMESS package [110] was used for geometry optimization.

### 3.2. Docking Setup

Docking simulations were performed with AutoDock Vina (version 1.1.2) software [111]. The docking search space for exploring ligand binding conformations around each representative A*β*_42_ structure was defined using a rectangular box centered at the center of mass of A*β*_42_ with a minimal distance of 12 Å from A*β*_42_ to the edges of the box. Therefore, depending on the size and shape of A*β*_42_ configuration, an optimized docking box was determined individually for each A*β*_42_. Each docking run generates nine optimal A*β*_42_-ligand bound conformations and overall, a total of 90,000 (10,000 × 9) poses were generated for each catechin compound. The different poses in each run are rank-ordered by the Vina score, a quantity that correlates with the binding free energy. The top-scoring pose in each run achieves the lowest free energy of binding in the complex.

Very recently, it has been shown that the correct pose (the pose with the lowest RMSD from the corresponding experimental pose) is usually predicted by Vina but sometimes, does not get the top score in the Vina ranking [112,113]. To avoid the problem and to capture the correct poses, it is recommended that except for the top-ranked pose, some important lower-ranked poses for each docking run should be identified and selected for post-docking analysis. For more discussions about ranking, see also [114,115,116,117]. For this purpose, the differences in the binding free energies between the top-ranked pose and lower-ranked poses were calculated for selected high-ranked modelled complexes for each docking run, ΔΔ*G_binding_* (= ΔG_top pose_ – ΔG_lower-ranked pose_). Different cutoff values for the ΔΔ*G_binding_* threshold (0.1, 0.2 and 0.3 kcal.mol^−1^) were used for selecting docking complexes, since the optimal selection of complexes is not known. For larger ΔΔ*G_binding_* cutoff values, more complexes were selected. For example, with the cutoff value of 0.1 kcal.mol^−1^, 17,431 (10,000 × 1 (top-ranked poses from each run) + 7431 (lower-ranked poses near top poses with the cutoff)) complexes were selected for docking of *EGCG*, while 29,923 and 42,833 complexes were selected with the cutoff values of 0.2 and 0.3 kcal.mol^−1^, respectively. The number of selected docking complexes for all ligands and the corresponding cutoff values of ΔΔ*G_binding_* are provided and labelled as “set-*n*” in Table 4. Since the results for different sets are very similar, we only present the results of set-1 (with the cutoff value of 0.1) and the results for the other two sets can be found in the Supporting Information. It should be emphasized that we employed the Vina ranking score just for selecting high-ranked docking poses for the structural analyses. The binding energies associated with these poses do not provide further insights.

### 3.3. MD Simulation Setup

All-atom MD simulations were performed on the peptide with five different ligands. The force field parameters for each ligand were created using the Antechamber program in the Ambertools19 package [118] and described by the General Amber Force Field (GAFF) [119] using AM1-BCC charges [120]. The Amberff99SB*-ILDNP force field [121] and the TIP3P water model [122] were adopted for the protein and water, respectively. Each protein was placed in a dodecahedral box such that the distance from the edges of the box to every atom in the protein was at least 1 nm and 150 mM of KCl was added to reproduce physiological conditions. Overall charge neutrality was preserved by adding 3 K^+^ counterions. The GROMACS 2016.3 [123]. package was used for all simulations. Each system was energy minimized using the method of steepest descents. This was followed by a pre-equilibration in the canonical ensemble, i.e., at constant particle number, volume, and temperature, for 100 ps. The Lennard–Jones potential was truncated using a shift function between 1.0 and 1.2 nm. Electrostatic interactions were calculated using the particle-mesh Ewald method (PME) [124,125] with a real space cutoff of 1.2 nm. The temperature was set to 310 K with the V-rescale algorithm [126] and pressure was kept at 1 atm using the Parrinello–Rahman barostat [127]. Bonds involving hydrogens were constrained using the linear constraint solver (P-LINCS) algorithm [128]. Pre-equilibration was followed by a production run of 3 μs with a time step of 2 fs for each of the five peptide-ligand systems.

## 4. Conclusions

In this work, binding of various well-known catechins present in green tea to the amyloid-*β* peptide (A*β*) has been predicted and analyzed. For this purpose, a computational pipeline in the framework of the ensemble docking strategy has been proposed in which a structurally heterogeneous ensemble of conformations of A*β*_42_ is used. The ensemble is generated by the Blockwise Excursion Sampling (BES) protocol [75,76] in which the conformational sampling is performed on the basis of many uncorrelated short-time MD simulations starting from different reasonable points of the accessible phase space.

It was observed that all green tea catechins compounds tended to interact with the aromatic residues through stacking and/or T-shaped interactions and, because of this, all compounds show a high tendency to interact with the hydrophobic region of A*β*_42_ spanning residues from Tyr10 to Phe20, the region with the highest number of the aromatic residues in full-length A*β*_42_. This region also encompasses the central hydrophobic core (CHC, residues 16–20) that, based on many experimental and computational studies, plays a key role in the aggregation process of A*β*_42_. Therefore, the docking results indicate that all studied ligands, especially *EGCG*, can act as potent inhibitors against amyloid aggregation by blocking the central hydrophobic core. Additionally, it has been suggested that both hydrophobic aromatic interactions and hydrogen bonding are crucial for the binding of catechins to A*β*_42_.

To evaluate the obtained findings in binding of catechin compounds to A*β*_42_, long multi-microsecond MD simulations were performed. It was shown that the present docking protocol is highly successful in identifying catechins’ binding sites in monomeric A*β*_42_, in agreement with previous MD simulations and some recent experimental observations for similar A*β*_42_–catechin complexes [28,77,78,79,83,84,85,87,93]. Finally, we suggested that our proposed pipeline with low computational cost in comparison with MD simulations is a suitable approach for high-throughput structure-based virtual screening of ligand libraries against the intrinsically disordered proteins (IDPs), such as A*β*. The execution of our proposed docking protocol for each ligand took up to a week using a standard Intel Core i7 desktop computer, while MD simulations for each ligand required, on average, approximately six weeks on a single compute node of the Compute Canada clusters, containing 24 CPU cores and 4 NVIDIA Tesla P100 GPUs.

## Figures and Tables

**Table 1:** The number of contacts between the amino acid residues of A*β*_42_ (For the full amino acid sequence of A*β*_42_, see Section 3.1) and each ligand for set-1 (as defined in Section 3.2) and the distance cutoff of 5.0 Å. The aromatic residues are in bold typeface and the three most favorable aromatic residue hotspots are highlighted in light blue.

*C*			*EC*			*ECG*			*EGC*			*EGCG*		
Residue	ID	Population	Residue	ID	Population	Residue	ID	Population	Residue	ID	Population	Residue	ID	Population
**PHE**	**19**	**6290**	**PHE**	**19**	**6194**	**PHE**	**19**	**7425**	**TYR**	**10**	**7107**	**TYR**	**10**	**8585**
**PHE**	**20**	**6242**	**PHE**	**20**	**6083**	**PHE**	**20**	**7304**	**PHE**	**19**	**7010**	**PHE**	**19**	**8302**
**TYR**	**10**	**6014**	**TYR**	**10**	**5967**	**TYR**	**10**	**7187**	**PHE**	**20**	**6931**	**PHE**	**20**	**8282**
GLN	15	5911	GLN	15	5800	GLN	15	7058	GLN	15	6792	GLN	15	8159
LYS	16	5586	LEU	17	5497	**HIS**	**14**	**6641**	**HIS**	**13**	**6456**	**HIS**	**13**	**7861**
LEU	17	5581	LYS	16	5456	**HIS**	**13**	**6620**	**HIS**	**14**	**6438**	**HIS**	**14**	**7760**
VAL	18	5423	VAL	18	5418	LEU	17	6578	LYS	16	6300	VAL	12	7712
**HIS**	**13**	**5386**	**HIS**	**14**	**5403**	VAL	18	6545	VAL	12	6254	LYS	16	7546
**HIS**	**14**	**5381**	**HIS**	**13**	**5375**	LYS	16	6545	LEU	17	6251	LEU	17	7538
VAL	12	5279	VAL	12	5214	VAL	12	6495	VAL	18	6212	VAL	18	7463
GLU	11	4785	GLU	11	4776	GLU	11	5988	GLU	11	5730	GLU	11	7189
ALA	21	4721	ALA	21	4671	ALA	21	5763	ALA	21	5459	ALA	21	6624
GLY	9	4225	GLY	9	4282	GLY	9	5452	GLY	9	5250	GLY	9	6552
GLU	22	4223	GLU	22	4244	SER	8	5246	SER	8	5198	SER	8	6389
SER	8	4122	SER	8	4177	ARG	5	5178	ARG	5	5144	ARG	5	6294
ARG	5	4075	ARG	5	4119	GLU	22	5176	GLU	22	5051	**HIS**	**6**	**6105**
**HIS**	**6**	**3934**	**HIS**	**6**	**3999**	**HIS**	**6**	**4978**	**HIS**	**6**	**4989**	GLU	22	5944
ASP	7	3715	ASP	7	3829	ASP	7	4792	ASP	7	4670	ASP	7	5906
VAL	24	3710	**PHE**	**4**	**3694**	**PHE**	**4**	**4623**	**PHE**	**4**	**4522**	**PHE**	**4**	**5621**
**PHE**	**4**	**3686**	VAL	24	3688	VAL	24	4612	VAL	24	4373	VAL	24	5316
ASP	23	3430	ASP	23	3414	ASP	23	4334	ASP	23	4127	ASP	23	4988
SER	26	3110	SER	26	3150	SER	26	3998	SER	26	3871	SER	26	4651
ASN	27	2975	ASN	27	3001	ASN	27	3808	ASN	27	3660	ASN	27	4429
GLY	25	2927	GLY	25	2952	GLY	25	3751	GLY	25	3598	GLY	25	4363
LYS	28	2766	LYS	28	2717	GLU	3	3548	GLU	3	3387	GLU	3	4314
GLU	3	2685	GLU	3	2676	LYS	28	3512	LYS	28	3259	LYS	28	4067
ILE	31	2473	ILE	31	2501	ALA	30	3044	ILE	31	2928	ALA	30	3506
ALA	30	2427	ALA	30	2458	ILE	31	3014	ALA	30	2910	ILE	31	3471
GLY	29	2310	GLY	29	2336	GLY	29	2903	GLY	29	2808	ALA	2	3358
ILE	32	2254	ILE	32	2298	ILE	32	2808	ILE	32	2689	GLY	29	3320
LEU	34	2110	LEU	34	2095	ALA	2	2753	ALA	2	2595	ILE	32	3261
ALA	2	2040	ALA	2	2068	LEU	34	2549	LEU	34	2397	LEU	34	2977
MET	35	1875	MET	35	1889	MET	35	2415	MET	35	2185	MET	35	2792
VAL	36	1822	VAL	36	1810	VAL	36	2284	VAL	36	2104	VAL	36	2595
GLY	33	1672	GLY	33	1729	GLY	33	2139	GLY	33	2004	ASP	1	2452
VAL	39	1559	VAL	39	1569	VAL	39	2038	ASP	1	1872	GLY	33	2451
ASP	1	1416	ASP	1	1414	ASP	1	2008	VAL	39	1867	VAL	39	2423
VAL	40	1392	GLY	37	1412	VAL	40	1824	GLY	37	1635	GLY	38	2126
GLY	37	1373	VAL	40	1353	ILE	41	1805	VAL	40	1620	ILE	41	2103
ILE	41	1372	GLY	38	1346	GLY	37	1792	GLY	38	1591	VAL	40	2094
GLY	38	1305	ILE	41	1310	GLY	38	1761	ILE	41	1557	GLY	37	2090
ALA	42	870	ALA	42	837	ALA	42	1202	ALA	42	1060	ALA	42	1466

**Table 2:** Average A*β_42_* end-to-end distance (D1–A42) and distances between *EGCG* and Y10, F19, F20 residues (the amino acid sequence is provided in the caption of Figure 7, in Section 3.1). Standard deviations are provided in parentheses.

	Distance (nm)				
	*C*	*EC*	*ECG*	*EGC*	*EGCG*
**end-to-end**	1.10 (0.66)	1.69 (0.73)	1.30 (0.48)	1.77 (0.60)	1.54 (0.59)
**Ligand∙∙∙Tyr10**	1.08 (0.45)	0.47 (0.40)	0.54 (0.18)	0.29 (0.08)	0.70 (0.22)
**Ligand∙∙∙Phe19**	0.97 (0.54)	0.95 (0.51)	0.55 (0.25)	0.50 (0.20)	0.40 (0.23)
**Ligand∙∙∙Phe20**	0.74 (0.53)	0.72 (0.39)	0.44 (0.24)	0.44 (0.20)	0.36 (0.26)

**Table 3:** Average number of hydrogen bonds (donor∙∙∙acceptor ≤ 0.35 nm and α (∠(hydrogen–donor–acceptor) ≤ 30°). Standard deviations are provided in parentheses.

	Number of Hydrogen Bonds (SD)				
	*C*	*EC*	*ECG*	*EGC*	*EGCG*
**A*β*∙∙∙Ligand**	2 (2)	2 (1)	4 (2)	4 (1)	5 (2)
**A*β*∙∙∙Solvent**	109 (17)	114 (20)	108 (21)	114 (21)	112 (8)
**Ligand∙∙∙Solvent**	6 (2)	5 (3)	7 (4)	5 (3)	7 (3)

**Table 4:** The number of selected docking complexes for each ligand with the different ΔΔ*G_binding_* cutoffs.

	ΔΔ*G_binding_* (kcal.mol^−1^) Cutoff	*C*	*EC*	*EGC*	*ECG*	*EGCG*
Set-1	0.1	13,495	13,478	15,646	15,027	17,431
Set-2	0.2	20,768	20,722	24,811	23,327	29,923
Set-3	0.3	30,544	30,712	35,758	34,105	42,833

## Data Availability

GAMESS package and AutoDock Vina (version 1.1.2) were used under a free academic license for ligands preparation and docking simulations. Produced and analyzed data are available upon request. MD simulations were performed using GROMACS 2016.3. Produced and analyzed data are available upon request.

## Supplementary Materials

The following supporting information can be downloaded at: https://www.mdpi.com/article/10.3390/ijms24098161/s1, Reference [129] are cited in the Supplementary Materials.
