# Supplementary material for: Identification of Catechins’ Binding Sites in Monomeric Aβ42 through Ensemble Docking and MD Simulations
Source: Int J Mol Sci. 2023 May 3;24(9):8161. doi: 10.3390/ijms24098161 (PMC10179585; doi:10.3390/ijms24098161)
Supplement: Supplementary file 1 [file ijms-24-08161-s001.zip › ijms-2311972-supplementary.pdf]

Supporting Information

Identification of Catechins' Binding Sites in Monomeric Aβ_{42} through Ensemble Docking and MD Simulations

Rohoullah Firouzi[a*], Shahin Sowlati-Hashjin[b], Cecilia Chávez-García[(c,d)], Mitra Ashouri[e], Mohammad Hossein Karimi-Jafari[f], Mikko Karttunen[(c,d,g)*]

[(a)] Department of Physical Chemistry, Chemistry and Chemical Engineering Research Center of Iran, Tehran, Iran. [(b)] Institute of Biomedical Engineering, University of Toronto, Toronto, Ontario, M5S 3G9, Canada. [(c)] Department of Chemistry, The University of Western Ontario, 1151 Richmond Street, London, Ontario N6A 5B7, Canada. [(d)] The Centre of Advanced Materials and Biomaterials Research, The University of Western Ontario, 1151 Richmond Street, London, Ontario N6A 5B7, Canada. [(e)] Department of Physical Chemistry, School of Chemistry, College of Science, University of Tehran, Tehran, Iran. [(f)] Department of Bioinformatics, Institute of Biochemistry and Biophysics, University of Tehran, Tehran, Iran. [(g)] Department of Physics and Astronomy, The University of Western Ontario, 1151 Richmond Street, London, Ontario N6A 3K7, Canada.

Table S1. The number of contacts between the amino acid residues (the amino acid sequence is provided in the caption of **Figure 1**) of Aβ_{42} and the *C* ligand of set-1 (13,495 complexes, **Table 1**) with five different distance cutoffs as indicated. The aromatic residues are in bold typeface and the three most favorable aromatic residue hotspots are highlighted in light teal.

**3.0 Å**			**3.5 Å**			**4.0 Å**			**4.5 Å**			**5.0 Å**		
**Residue**	**ID**	**Population**	**Residue**	**ID**	**Population**	**Residue**	**ID**	**Population**	**Residue**	**ID**	**Population**	**Residue**	**ID**	**Population**
**PHE**	**19**	**4455**	**PHE**	**19**	**5160**	**PHE**	**19**	**5510**	**PHE**	**19**	**5853**	**PHE**	**19**	**6290**
**TYR**	**10**	**4263**	**TYR**	**10**	**4916**	**PHE**	**20**	**5297**	**PHE**	**20**	**5739**	**PHE**	**20**	**6242**
**PHE**	**20**	**4138**	**PHE**	**20**	**4884**	**TYR**	**10**	**5267**	**TYR**	**10**	**5603**	**TYR**	**10**	**6014**
GLN	15	3963	GLN	15	4602	GLN	15	4987	GLN	15	5419	GLN	15	5911
LYS	16	3745	LYS	16	4220	LYS	16	4611	LYS	16	5040	LYS	16	5586
LEU	17	3472	LEU	17	4053	LEU	17	4515	LEU	17	4999	LEU	17	5581
**HIS**	**13**	**3402**	**HIS**	**13**	**4030**	**HIS**	**13**	**4441**	**HIS**	**13**	**4885**	VAL	18	5423
**HIS**	**14**	**3338**	**HIS**	**14**	**4000**	**HIS**	**14**	**4412**	**HIS**	**14**	**4850**	**HIS**	**13**	**5386**
VAL	18	3246	VAL	18	3938	VAL	18	4371	VAL	18	4838	**HIS**	**14**	**5381**
VAL	12	3027	VAL	12	3713	VAL	12	4171	VAL	12	4661	VAL	12	5279
GLU	11	2926	GLU	11	3418	GLU	11	3797	GLU	11	4265	GLU	11	4785
ARG	5	2642	ALA	21	3237	ALA	21	3688	ALA	21	4169	ALA	21	4721
**PHE**	**4**	**2628**	GLU	22	3061	GLU	22	3411	GLU	22	3776	GLY	9	4225
ALA	21	2617	ARG	5	3054	ARG	5	3360	GLY	9	3718	GLU	22	4223
GLU	22	2587	**HIS**	**6**	**3038**	**HIS**	**6**	**3321**	ARG	5	3712	SER	8	4122
**HIS**	**6**	**2530**	**PHE**	**4**	**3026**	GLY	9	3262	SER	8	3632	ARG	5	4075
SER	8	2337	SER	8	2869	SER	8	3258	**HIS**	**6**	**3615**	**HIS**	**6**	**3934**
GLY	9	2265	GLY	9	2866	**PHE**	**4**	**3255**	**PHE**	**4**	**3452**	ASP	7	3715
VAL	24	2154	ASP	7	2587	VAL	24	2931	VAL	24	3289	VAL	24	3710
ASP	7	2140	VAL	24	2587	ASP	7	2904	ASP	7	3265	**PHE**	**4**	**3686**
ASP	23	1905	ASP	23	2327	ASP	23	2602	ASP	23	2985	ASP	23	3430
ASN	27	1895	ASN	27	2190	ASN	27	2393	SER	26	2721	SER	26	3110
SER	26	1738	SER	26	2076	SER	26	2374	ASN	27	2667	ASN	27	2975
GLU	3	1705	GLU	3	2037	GLU	3	2256	GLY	25	2514	GLY	25	2927
LYS	28	1684	LYS	28	1957	GLY	25	2186	GLU	3	2463	LYS	28	2766
GLY	25	1459	GLY	25	1879	LYS	28	2172	LYS	28	2433	GLU	3	2685
ILE	31	1452	ILE	31	1711	ILE	31	1916	ILE	31	2172	ILE	31	2473
ALA	30	1327	ALA	30	1613	ALA	30	1844	ALA	30	2101	ALA	30	2427
ILE	32	1291	ILE	32	1538	ILE	32	1749	GLY	29	1981	GLY	29	2310
LEU	34	1225	GLY	29	1489	GLY	29	1719	ILE	32	1979	ILE	32	2254
ALA	2	1169	LEU	34	1461	ALA	2	1641	ALA	2	1843	LEU	34	2110
GLY	29	1151	ALA	2	1434	LEU	34	1637	LEU	34	1842	ALA	2	2040
VAL	36	1049	VAL	36	1264	MET	35	1449	MET	35	1652	MET	35	1875
MET	35	966	MET	35	1245	VAL	36	1415	VAL	36	1582	VAL	36	1822
GLY	33	914	GLY	33	1148	GLY	33	1297	GLY	33	1471	GLY	33	1672
VAL	39	854	VAL	39	1048	VAL	39	1193	VAL	39	1342	VAL	39	1559
VAL	40	832	VAL	40	982	ASP	1	1116	ASP	1	1265	ASP	1	1416
ASP	1	806	ASP	1	977	VAL	40	1084	VAL	40	1225	VAL	40	1392
ILE	41	771	ILE	41	948	ILE	41	1074	ILE	41	1213	GLY	37	1373
GLY	37	648	GLY	37	815	GLY	37	984	GLY	37	1135	ILE	41	1372
GLY	38	643	GLY	38	814	GLY	38	953	GLY	38	1092	GLY	38	1305
ALA	42	468	ALA	42	580	ALA	42	674	ALA	42	764	ALA	42	870

Table S2. The number of contacts between the amino acid residues (the amino acid sequence is provided in the caption of **Figure 1**) of Aβ_{42} and the *EC* ligand for set-1 (13,478 complexes, **Table 1**) with five different distance cutoffs as indicated. The aromatic residues are in bold typeface and the three most favorable aromatic residue hotspots are highlighted in light teal.

**3.0 Å**			**3.5 Å**			**4.0 Å**			**4.5 Å**			**5.0 Å**		
**Residue**	**ID**	**Population**	**Residue**	**ID**	**Population**	**Residue**	**ID**	**Population**	**Residue**	**ID**	**Population**	**Residue**	**ID**	**Population**
**PHE**	**19**	**4264**	**PHE**	**19**	**4971**	**PHE**	**19**	**5355**	**PHE**	**19**	**5741**	**PHE**	**19**	**6194**
**TYR**	**10**	**4181**	**TYR**	**10**	**4899**	**TYR**	**10**	**5273**	**TYR**	**10**	**5620**	**PHE**	**20**	**6083**
**PHE**	**20**	**4008**	**PHE**	**20**	**4776**	**PHE**	**20**	**5190**	**PHE**	**20**	**5617**	**TYR**	**10**	**5967**
GLN	15	3947	GLN	15	4527	GLN	15	4907	GLN	15	5308	GLN	15	5800
LYS	16	3643	LYS	16	4103	LYS	16	4483	LYS	16	4954	LEU	17	5497
**HIS**	**13**	**3433**	**HIS**	**13**	**4070**	**HIS**	**13**	**4459**	**HIS**	**13**	**4892**	LYS	16	5456
LEU	17	3355	LEU	17	3963	LEU	17	4403	LEU	17	4890	VAL	18	5418
**HIS**	**14**	**3324**	**HIS**	**14**	**3959**	**HIS**	**14**	**4377**	**HIS**	**14**	**4834**	**HIS**	**14**	**5403**
VAL	18	3255	VAL	18	3878	VAL	18	4322	VAL	18	4820	**HIS**	**13**	**5375**
VAL	12	3113	VAL	12	3740	VAL	12	4186	VAL	12	4660	VAL	12	5214
GLU	11	2890	GLU	11	3391	GLU	11	3789	GLU	11	4250	GLU	11	4776
ARG	5	2802	ALA	21	3299	ALA	21	3718	ALA	21	4169	ALA	21	4671
ALA	21	2678	ARG	5	3237	ARG	5	3500	ARG	5	3817	GLY	9	4282
GLU	22	2648	**HIS**	**6**	**3112**	GLU	22	3432	GLU	22	3812	GLU	22	4244
**HIS**	**6**	**2624**	GLU	22	3099	**HIS**	**6**	**3364**	GLY	9	3772	SER	8	4177
**PHE**	**4**	**2551**	**PHE**	**4**	**2991**	GLY	9	3325	SER	8	3719	ARG	5	4119
SER	8	2461	GLY	9	2896	SER	8	3275	**HIS**	**6**	**3671**	**HIS**	**6**	**3999**
GLY	9	2279	SER	8	2895	**PHE**	**4**	**3242**	**PHE**	**4**	**3467**	ASP	7	3829
ASP	7	2218	ASP	7	2659	ASP	7	2982	ASP	7	3355	**PHE**	**4**	**3694**
VAL	24	2094	VAL	24	2520	VAL	24	2862	VAL	24	3214	VAL	24	3688
ASN	27	1894	ASP	23	2283	ASP	23	2582	ASP	23	2934	ASP	23	3414
ASP	23	1871	ASN	27	2182	SER	26	2442	SER	26	2769	SER	26	3150
SER	26	1831	SER	26	2158	ASN	27	2419	ASN	27	2695	ASN	27	3001
GLU	3	1702	GLU	3	2024	GLY	25	2259	GLY	25	2579	GLY	25	2952
LYS	28	1617	GLY	25	1972	GLU	3	2238	GLU	3	2431	LYS	28	2717
GLY	25	1571	LYS	28	1891	LYS	28	2116	LYS	28	2395	GLU	3	2676
ILE	31	1473	ILE	31	1753	ILE	31	1939	ILE	31	2201	ILE	31	2501
ALA	30	1373	ALA	30	1666	ALA	30	1871	ALA	30	2123	ALA	30	2458
ILE	32	1307	ILE	32	1582	ILE	32	1785	GLY	29	2049	GLY	29	2336
LEU	34	1250	GLY	29	1505	GLY	29	1742	ILE	32	2010	ILE	32	2298
GLY	29	1179	LEU	34	1451	ALA	2	1634	ALA	2	1837	LEU	34	2095
ALA	2	1128	ALA	2	1427	LEU	34	1602	LEU	34	1831	ALA	2	2068
VAL	36	1014	VAL	36	1227	MET	35	1414	MET	35	1630	MET	35	1889
MET	35	971	MET	35	1207	VAL	36	1371	VAL	36	1575	VAL	36	1810
GLY	33	967	GLY	33	1185	GLY	33	1332	GLY	33	1522	GLY	33	1729
VAL	39	856	VAL	39	1043	VAL	39	1183	VAL	39	1344	VAL	39	1569
ASP	1	793	ASP	1	981	ASP	1	1125	ASP	1	1260	ASP	1	1414
VAL	40	764	VAL	40	918	VAL	40	1043	VAL	40	1191	GLY	37	1412
ILE	41	724	ILE	41	890	GLY	37	1030	GLY	37	1191	VAL	40	1353
GLY	37	686	GLY	37	868	ILE	41	1023	ILE	41	1170	GLY	38	1346
GLY	38	608	GLY	38	803	GLY	38	926	GLY	38	1112	ILE	41	1310
ALA	42	446	ALA	42	534	ALA	42	631	ALA	42	734	ALA	42	837

Table S3. The number of contacts between the amino acid residues (the amino acid sequence is provided in the caption of **Figure 1**) of Aβ_{42} and the *ECG* ligand for set-1 (15,027 complexes, **Table 1**) with five different distance cutoffs as indicated. The aromatic residues are in bold typeface and the three most favorable aromatic residue hotspots are highlighted in light teal.

**3.0 Å**			**3.5 Å**			**4.0 Å**			**4.5 Å**			**5.0 Å**		
**Residue**	**ID**	**Population**	**Residue**	**ID**	**Population**	**Residue**	**ID**	**Population**	**Residue**	**ID**	**Population**	**Residue**	**ID**	**Population**
**PHE**	**19**	**5225**	**PHE**	**19**	**6050**	**PHE**	**19**	**6491**	**PHE**	**19**	**6953**	**PHE**	**19**	**7425**
**TYR**	**10**	**5192**	**TYR**	**10**	**5937**	**TYR**	**10**	**6338**	**PHE**	**20**	**6751**	**PHE**	**20**	**7304**
**PHE**	**20**	**4974**	**PHE**	**20**	**5792**	**PHE**	**20**	**6266**	**TYR**	**10**	**6743**	**TYR**	**10**	**7187**
GLN	15	4954	GLN	15	5629	GLN	15	6025	GLN	15	6506	GLN	15	7058
LYS	16	4384	**HIS**	**13**	**5062**	**HIS**	**13**	**5589**	**HIS**	**13**	**6053**	**HIS**	**14**	**6641**
**HIS**	**13**	**4322**	LYS	16	4985	LYS	16	5467	**HIS**	**14**	**6017**	**HIS**	**13**	**6620**
**HIS**	**14**	**4218**	**HIS**	**14**	**4944**	**HIS**	**14**	**5459**	LYS	16	5968	LEU	17	6578
LEU	17	4134	LEU	17	4838	LEU	17	5332	LEU	17	5901	VAL	18	6545
VAL	18	4081	VAL	18	4820	VAL	18	5322	VAL	18	5877	LYS	16	6545
VAL	12	4029	VAL	12	4770	VAL	12	5305	VAL	12	5857	VAL	12	6495
GLU	11	3786	GLU	11	4406	GLU	11	4862	GLU	11	5393	GLU	11	5988
ARG	5	3652	ALA	21	4112	ALA	21	4620	ALA	21	5151	ALA	21	5763
ALA	21	3416	ARG	5	4101	ARG	5	4460	GLY	9	4876	GLY	9	5452
GLU	22	3332	GLU	22	3894	GLY	9	4328	ARG	5	4796	SER	8	5246
**HIS**	**6**	**3297**	SER	8	3889	SER	8	4312	SER	8	4712	ARG	5	5178
SER	8	3275	**HIS**	**6**	**3882**	GLU	22	4260	GLU	22	4690	GLU	22	5176
**PHE**	**4**	**3272**	GLY	9	3830	**HIS**	**6**	**4245**	**HIS**	**6**	**4610**	**HIS**	**6**	**4978**
GLY	9	3080	**PHE**	**4**	**3787**	**PHE**	**4**	**4088**	**PHE**	**4**	**4357**	ASP	7	4792
ASP	7	2965	ASP	7	3563	ASP	7	3917	ASP	7	4315	**PHE**	**4**	**4623**
VAL	24	2712	VAL	24	3259	VAL	24	3632	VAL	24	4062	VAL	24	4612
ASP	23	2550	ASP	23	3042	ASP	23	3404	ASP	23	3805	ASP	23	4334
ASN	27	2459	ASN	27	2851	SER	26	3202	SER	26	3560	SER	26	3998
SER	26	2413	SER	26	2848	ASN	27	3116	ASN	27	3406	ASN	27	3808
GLU	3	2320	GLU	3	2752	GLU	3	3025	GLY	25	3273	GLY	25	3751
LYS	28	2143	GLY	25	2527	GLY	25	2890	GLU	3	3259	GLU	3	3548
GLY	25	2052	LYS	28	2468	LYS	28	2772	LYS	28	3153	LYS	28	3512
ILE	31	1780	ILE	31	2114	ILE	31	2365	ILE	31	2642	ALA	30	3044
ALA	30	1659	ALA	30	2066	ALA	30	2325	ALA	30	2634	ILE	31	3014
ILE	32	1640	GLY	29	1937	GLY	29	2248	GLY	29	2533	GLY	29	2903
LEU	34	1568	ILE	32	1932	ALA	2	2194	ILE	32	2476	ILE	32	2808
ALA	2	1529	ALA	2	1919	ILE	32	2191	ALA	2	2470	ALA	2	2753
GLY	29	1507	LEU	34	1841	LEU	34	2027	LEU	34	2252	LEU	34	2549
VAL	36	1310	VAL	36	1589	MET	35	1803	MET	35	2060	MET	35	2415
MET	35	1251	MET	35	1553	VAL	36	1786	VAL	36	2018	VAL	36	2284
ASP	1	1192	GLY	33	1436	GLY	33	1646	GLY	33	1854	GLY	33	2139
GLY	33	1163	ASP	1	1435	ASP	1	1618	ASP	1	1805	VAL	39	2038
VAL	39	1115	VAL	39	1347	VAL	39	1572	VAL	39	1785	ASP	1	2008
ILE	41	1046	VAL	40	1267	VAL	40	1424	ILE	41	1622	VAL	40	1824
VAL	40	1043	ILE	41	1234	ILE	41	1420	VAL	40	1604	ILE	41	1805
GLY	37	848	GLY	37	1094	GLY	37	1300	GLY	37	1505	GLY	37	1792
GLY	38	836	GLY	38	1073	GLY	38	1272	GLY	38	1477	GLY	38	1761
ALA	42	656	ALA	42	829	ALA	42	948	ALA	42	1082	ALA	42	1202

Table S4. The number of contacts between the amino acid residues (the amino acid sequence is provided in the caption of **Figure 1**) of Aβ_{42} and the *EGC* ligand for set-1 (15,646 complexes, **Table 1**) with five different distance cutoffs as indicated. The aromatic residues are in bold typeface and the three most favorable aromatic residue hotspots are highlighted in light teal.

3.0 Å			3.5 Å			4.0 Å			4.5 Å			5.0 Å		
Residue	ID	Population	Residue	ID	Population	Residue	ID	Population	Residue	ID	Population	Residue	ID	Population
**TYR**	**10**	**5045**	**TYR**	**10**	**5821**	**TYR**	**10**	**6228**	**TYR**	**10**	**6643**	**TYR**	**10**	**7107**
**PHE**	**19**	**4812**	**PHE**	**19**	**5579**	**PHE**	**19**	**5989**	**PHE**	**19**	**6443**	**PHE**	**19**	**7010**
GLN	15	4686	**PHE**	**20**	**5397**	**PHE**	**20**	**5873**	**PHE**	**20**	**6376**	**PHE**	**20**	**6931**
**PHE**	**20**	**4577**	GLN	15	5325	GLN	15	5777	GLN	15	6236	GLN	15	6792
**HIS**	**13**	**4194**	**HIS**	**13**	**4938**	**HIS**	**13**	**5403**	**HIS**	**13**	**5875**	**HIS**	**13**	**6456**
LYS	16	4094	**HIS**	**14**	**4787**	**HIS**	**14**	**5262**	**HIS**	**14**	**5824**	**HIS**	**14**	**6438**
**HIS**	**14**	**4085**	LYS	16	4649	LYS	16	5108	LYS	16	5665	LYS	16	6300
LEU	17	3818	LEU	17	4491	LEU	17	5014	VAL	12	5588	VAL	12	6254
VAL	12	3747	VAL	12	4463	VAL	12	5000	LEU	17	5571	LEU	17	6251
VAL	18	3739	VAL	18	4417	VAL	18	4910	VAL	18	5514	VAL	18	6212
GLU	11	3570	GLU	11	4095	GLU	11	4554	GLU	11	5115	GLU	11	5730
ARG	5	3505	ARG	5	4024	ARG	5	4408	ALA	21	4895	ALA	21	5459
**HIS**	**6**	**3346**	**HIS**	**6**	**3899**	ALA	21	4339	ARG	5	4776	GLY	9	5250
GLU	22	3201	ALA	21	3839	**HIS**	**6**	**4232**	GLY	9	4640	SER	8	5198
ALA	21	3132	GLU	22	3712	SER	8	4130	**HIS**	**6**	**4601**	ARG	5	5144
**PHE**	**4**	**3114**	SER	8	3654	GLU	22	4117	SER	8	4588	GLU	22	5051
SER	8	3062	**PHE**	**4**	**3601**	GLY	9	4093	GLU	22	4553	**HIS**	**6**	**4989**
GLY	9	2901	GLY	9	3593	**PHE**	**4**	**3903**	**PHE**	**4**	**4202**	ASP	7	4670
ASP	7	2810	ASP	7	3312	ASP	7	3698	ASP	7	4123	**PHE**	**4**	**4522**
VAL	24	2480	VAL	24	2955	VAL	24	3331	VAL	24	3806	VAL	24	4373
ASN	27	2348	ASP	23	2796	ASP	23	3113	ASP	23	3558	ASP	23	4127
ASP	23	2341	SER	26	2710	SER	26	3041	SER	26	3430	SER	26	3871
SER	26	2283	ASN	27	2683	ASN	27	2937	ASN	27	3260	ASN	27	3660
GLU	3	2180	GLU	3	2599	GLU	3	2828	GLY	25	3151	GLY	25	3598
GLY	25	1904	GLY	25	2383	GLY	25	2753	GLU	3	3094	GLU	3	3387
LYS	28	1903	LYS	28	2235	LYS	28	2489	LYS	28	2860	LYS	28	3259
ILE	31	1689	ILE	31	1987	ILE	31	2239	ILE	31	2544	ILE	31	2928
ALA	30	1618	ALA	30	1961	ALA	30	2203	ALA	30	2516	ALA	30	2910
GLY	29	1510	GLY	29	1867	GLY	29	2139	GLY	29	2443	GLY	29	2808
ILE	32	1502	ILE	32	1826	ALA	2	2086	ILE	32	2347	ILE	32	2689
ALA	2	1433	ALA	2	1826	ILE	32	2057	ALA	2	2336	ALA	2	2595
LEU	34	1412	LEU	34	1638	LEU	34	1851	LEU	34	2110	LEU	34	2397
VAL	36	1166	VAL	36	1412	MET	35	1608	MET	35	1849	MET	35	2185
MET	35	1135	GLY	33	1385	VAL	36	1608	VAL	36	1840	VAL	36	2104
GLY	33	1115	MET	35	1379	GLY	33	1547	GLY	33	1767	GLY	33	2004
ASP	1	1059	ASP	1	1274	ASP	1	1457	ASP	1	1664	ASP	1	1872
VAL	39	1027	VAL	39	1236	VAL	39	1419	VAL	39	1618	VAL	39	1867
VAL	40	901	VAL	40	1077	VAL	40	1221	VAL	40	1407	GLY	37	1635
ILE	41	885	ILE	41	1060	ILE	41	1218	ILE	41	1403	VAL	40	1620
GLY	37	785	GLY	37	1014	GLY	37	1170	GLY	38	1365	GLY	38	1591
GLY	38	770	GLY	38	1009	GLY	38	1160	GLY	37	1360	ILE	41	1557
ALA	42	547	ALA	42	669	ALA	42	790	ALA	42	921	ALA	42	1060

Table S5. The number of contacts between the amino acid residues (the amino acid sequence is provided in the caption of **Figure 1**) of Aβ_{42} and the *EGCG* ligand for set-1 (17,431 complexes, **Table 1**) with five different distance cutoffs as indicated. The aromatic residues are in bold typeface and the three most favorable aromatic residue hotspots are highlighted in light teal.

3.0 Å			3.5 Å			4.0 Å			4.5 Å			5.0 Å		
Residue	ID	Population	Residue	ID	Population	Residue	ID	Population	Residue	ID	Population	Residue	ID	Population
**TYR**	**10**	**6305**	**TYR**	**10**	**7109**	**TYR**	**10**	**7572**	**TYR**	**10**	**8059**	**TYR**	**10**	**8585**
**PHE**	**19**	**5892**	**PHE**	**19**	**6734**	**PHE**	**19**	**7225**	**PHE**	**19**	**7758**	**PHE**	**19**	**8302**
GLN	15	5757	GLN	15	6495	**PHE**	**20**	**7012**	**PHE**	**20**	**7648**	**PHE**	**20**	**8282**
**PHE**	**20**	**5580**	**PHE**	**20**	**6450**	GLN	15	6960	GLN	15	7545	GLN	15	8159
**HIS**	**13**	**5184**	**HIS**	**13**	**6020**	**HIS**	**13**	**6604**	**HIS**	**13**	**7189**	**HIS**	**13**	**7861**
LYS	16	5071	**HIS**	**14**	**5811**	**HIS**	**14**	**6400**	**HIS**	**14**	**7066**	**HIS**	**14**	**7760**
**HIS**	**14**	**4997**	LYS	16	5723	VAL	12	6323	VAL	12	6989	VAL	12	7712
VAL	12	4802	VAL	12	5694	LYS	16	6253	LYS	16	6840	LYS	16	7546
LEU	17	4699	VAL	18	5496	VAL	18	6093	VAL	18	6733	LEU	17	7538
VAL	18	4685	LEU	17	5460	LEU	17	6017	LEU	17	6728	VAL	18	7463
GLU	11	4579	GLU	11	5311	GLU	11	5860	GLU	11	6492	GLU	11	7189
ARG	5	4483	ARG	5	5048	ARG	5	5437	ALA	21	5911	ALA	21	6624
**HIS**	**6**	**4115**	**HIS**	**6**	**4797**	ALA	21	5303	ARG	5	5886	GLY	9	6552
SER	8	4048	ALA	21	4779	GLY	9	5224	GLY	9	5856	SER	8	6389
**PHE**	**4**	**3974**	SER	8	4685	SER	8	5218	SER	8	5737	ARG	5	6294
ALA	21	3972	GLY	9	4605	**HIS**	**6**	**5212**	**HIS**	**6**	**5660**	**HIS**	**6**	**6105**
GLU	22	3883	**PHE**	**4**	**4525**	GLU	22	4907	GLU	22	5434	GLU	22	5944
GLY	9	3787	GLU	22	4479	**PHE**	**4**	**4901**	ASP	7	5281	ASP	7	5906
ASP	7	3646	ASP	7	4351	ASP	7	4781	**PHE**	**4**	**5269**	**PHE**	**4**	**5621**
VAL	24	3142	VAL	24	3745	VAL	24	4162	VAL	24	4662	VAL	24	5316
ASP	23	2959	ASP	23	3502	ASP	23	3880	ASP	23	4398	ASP	23	4988
SER	26	2873	SER	26	3372	SER	26	3766	SER	26	4208	SER	26	4651
ASN	27	2856	GLU	3	3328	GLU	3	3658	GLU	3	3988	ASN	27	4429
GLU	3	2819	ASN	27	3274	ASN	27	3584	ASN	27	3957	GLY	25	4363
LYS	28	2470	GLY	25	2920	GLY	25	3358	GLY	25	3820	GLU	3	4314
GLY	25	2373	LYS	28	2842	LYS	28	3226	LYS	28	3618	LYS	28	4067
ILE	31	2113	ILE	31	2453	ALA	2	2712	ALA	30	3080	ALA	30	3506
ALA	2	1937	ALA	2	2382	ILE	31	2712	ILE	31	3029	ILE	31	3471
ALA	30	1898	ALA	30	2371	ALA	30	2696	ALA	2	3005	ALA	2	3358
ILE	32	1863	GLY	29	2255	GLY	29	2597	GLY	29	2921	GLY	29	3320
GLY	29	1809	ILE	32	2239	ILE	32	2524	ILE	32	2866	ILE	32	3261
LEU	34	1780	LEU	34	2104	LEU	34	2330	LEU	34	2582	LEU	34	2977
ASP	1	1531	MET	35	1837	MET	35	2104	MET	35	2408	MET	35	2792
VAL	36	1516	VAL	36	1818	VAL	36	2047	VAL	36	2296	VAL	36	2595
MET	35	1469	ASP	1	1815	ASP	1	2027	ASP	1	2251	ASP	1	2452
VAL	39	1350	GLY	33	1661	GLY	33	1883	GLY	33	2135	GLY	33	2451
GLY	33	1348	VAL	39	1629	VAL	39	1872	VAL	39	2115	VAL	39	2423
ILE	41	1208	VAL	40	1455	ILE	41	1675	ILE	41	1885	GLY	38	2126
VAL	40	1205	ILE	41	1454	VAL	40	1649	VAL	40	1848	ILE	41	2103
GLY	38	1060	GLY	37	1340	GLY	37	1571	GLY	38	1787	VAL	40	2094
GLY	37	1040	GLY	38	1336	GLY	38	1541	GLY	37	1781	GLY	37	2090
ALA	42	818	ALA	42	1006	ALA	42	1147	ALA	42	1299	ALA	42	1466

Table S6. RMSD, R_g, and SASA of Aβ_{42} in the different systems (standard deviations in parentheses).

	Systems				
	**C**	**EC**	**ECG**	**EGC**	**EGCG**
**RMSD (nm)**	0.85 (0.12)	1.26 (0.09)	0.7 (0.09)	1.26 (0.17)	0.99 (0.14)
**R_g (nm)**	0.96 (0.09)	0.98 (0.07)	0.96 (0.07)	0.99 (0.07)	1.01 (0.06)
**SASA (nm^2)**	35.56 (3.19)	36.82 (2.53)	36.96 (2.03)	37.07 (1.91)	38.19 (1.89)

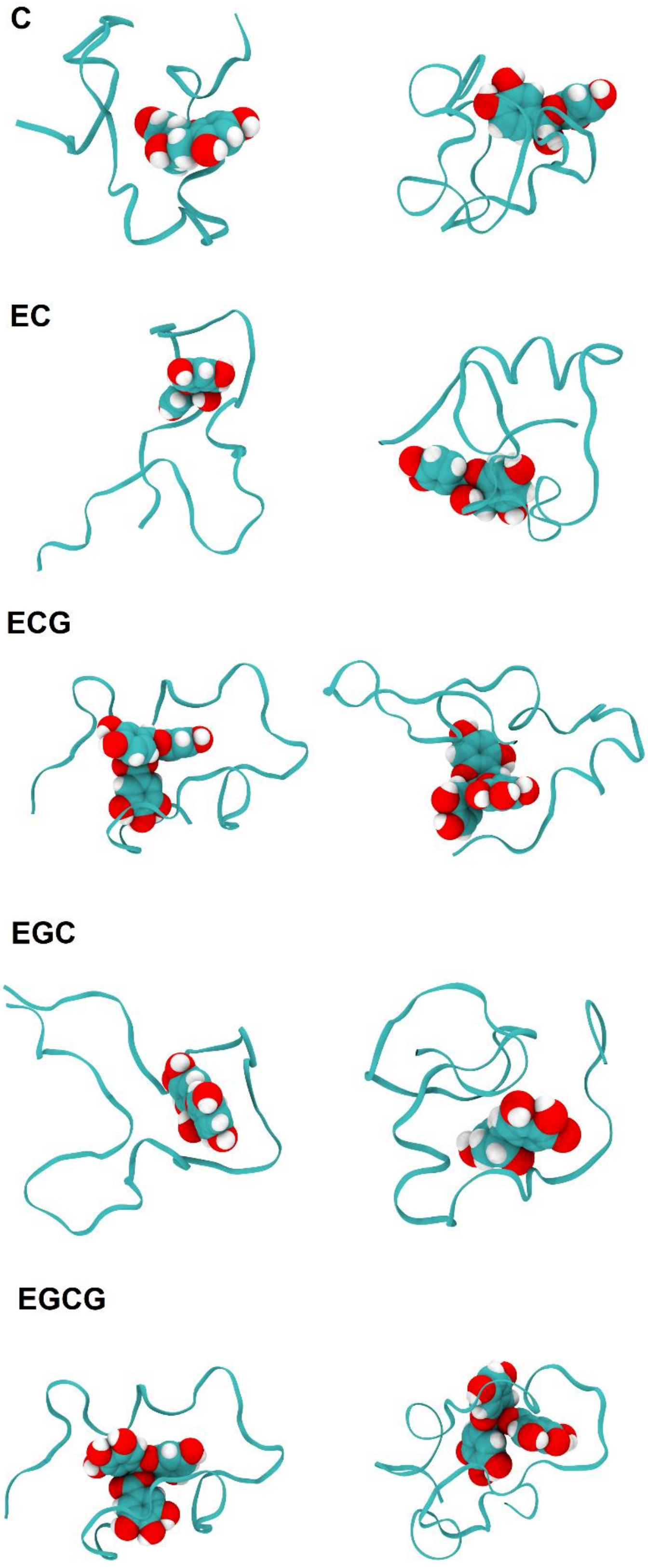

Figure S1. Initial (left) and final (right, at 3.0 μs) structures of Aβ_{42} in the presence of ligands. Aβ_{42} is shown using ribbons and the ligands using the van der Waals representation.

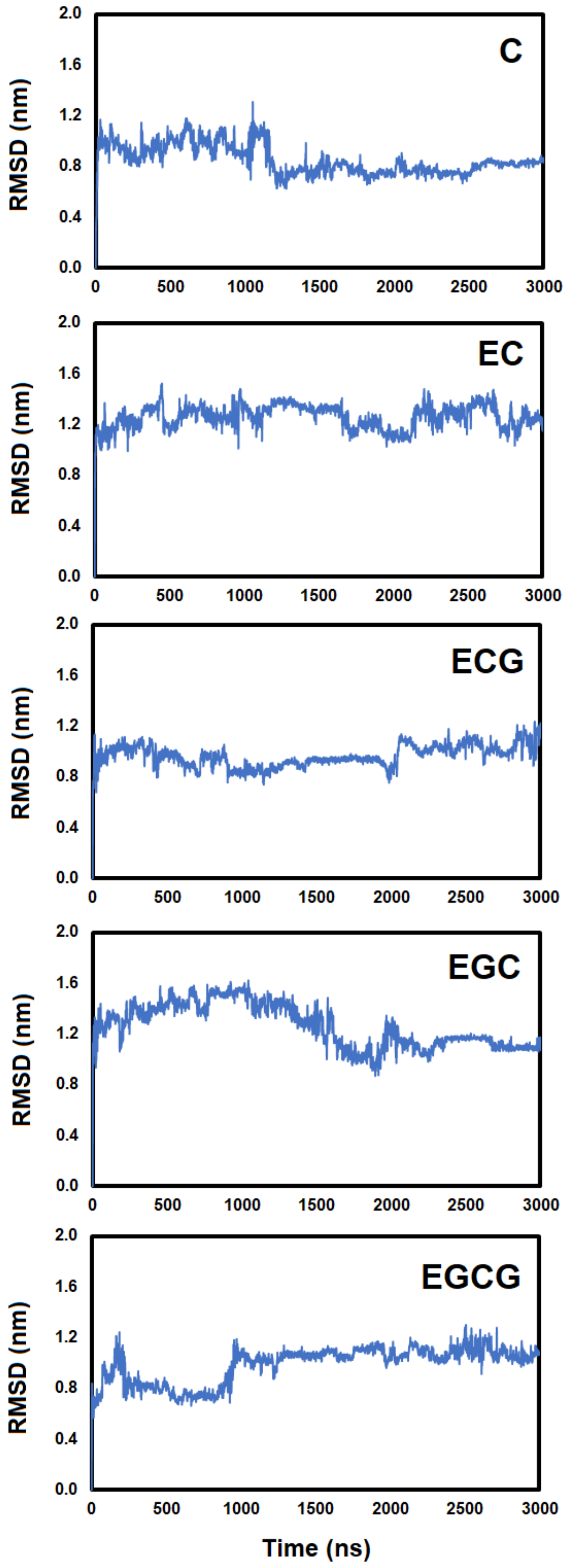

Figure S2. RMSD of the Aβ_{42} backbone in the presence of different ligands.

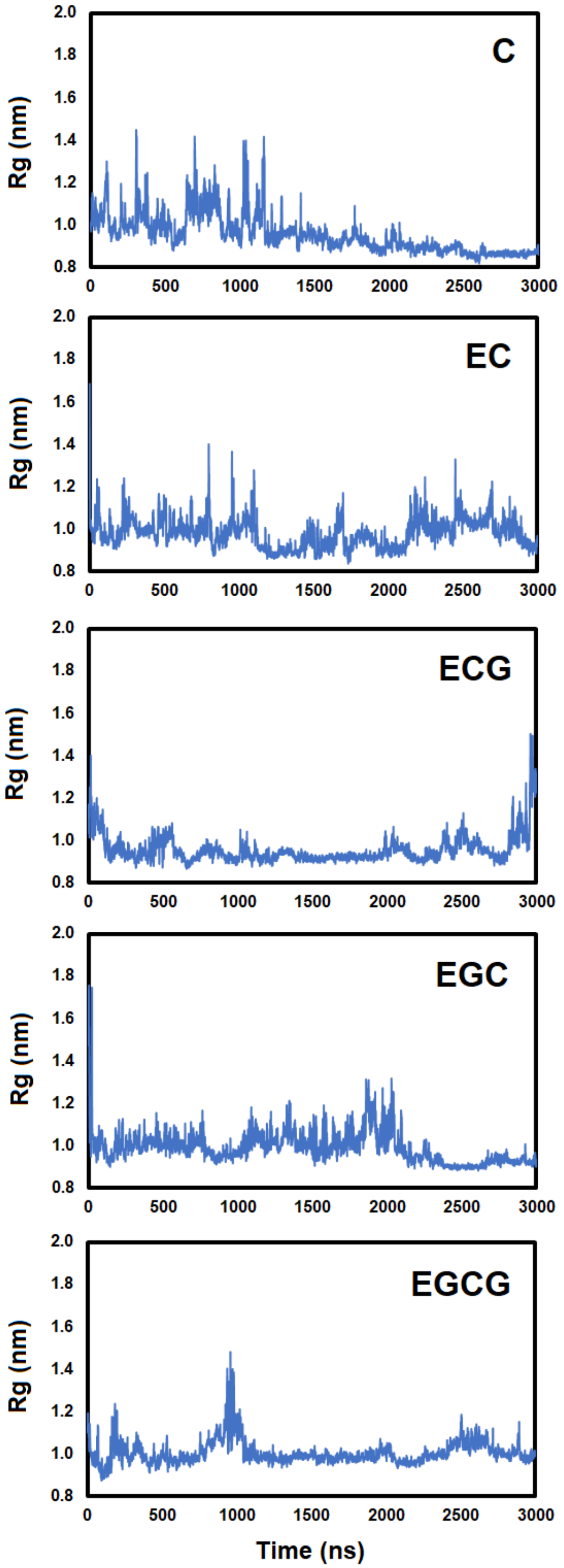

Figure S3. Radius of gyration of the Aβ_{42} backbone in the presence of different ligands.

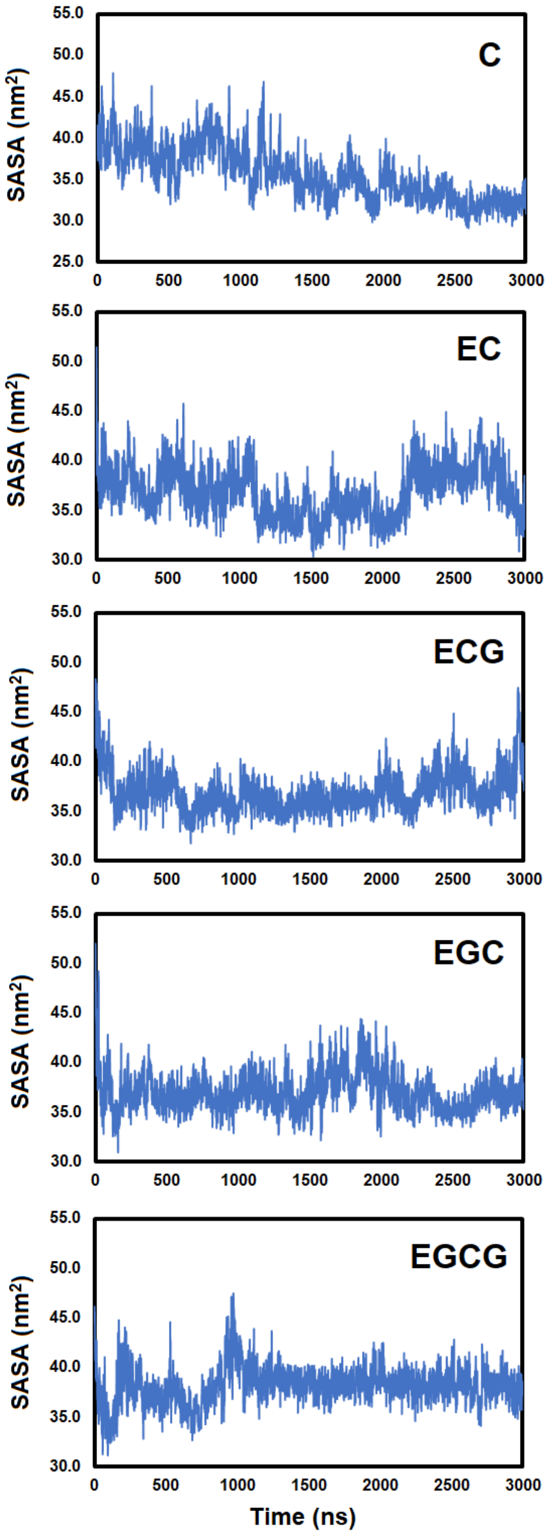

Figure S4. Solvent accessible surface area of the Aβ_{42} backbone in the presence of different ligands. SASA was calculated based on the numerical Double Cubic Lattice Method (DCLM)[1] as implemented in the GROMACS package using 1 ns intervals.

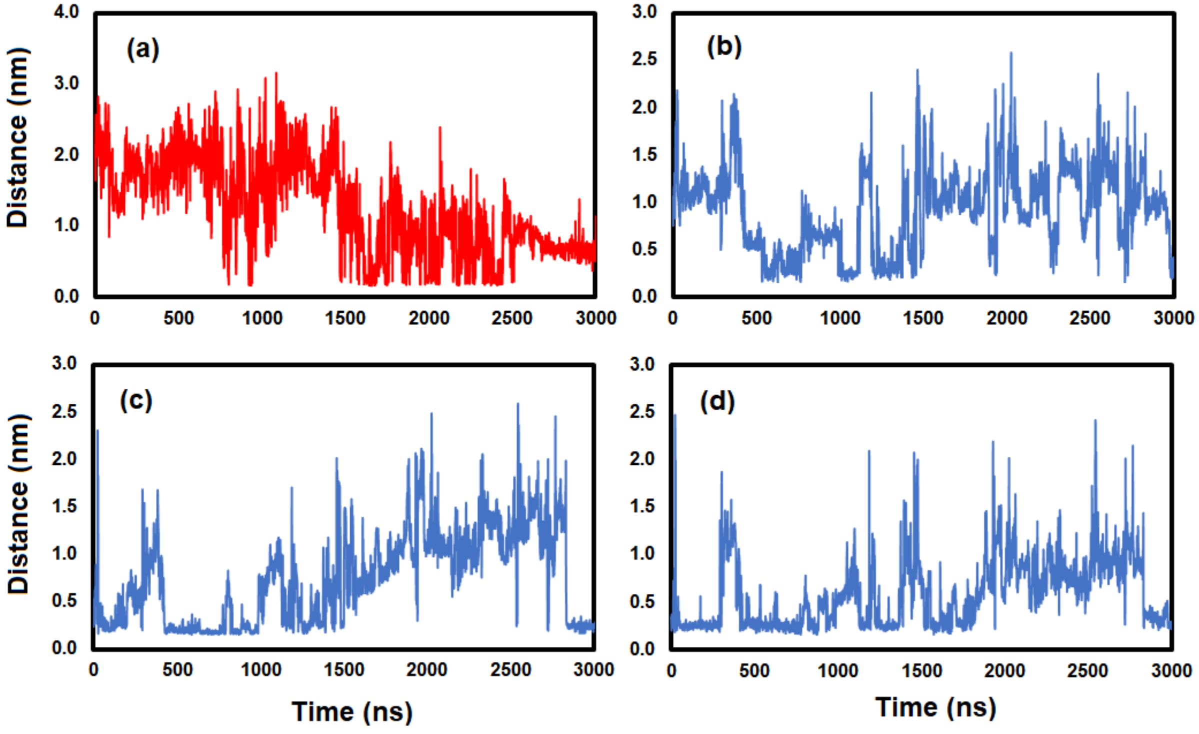

Figure S5. (a) Average Aβ_{42} end-to-end distance (D1–A42) and distances between **C** and (b) Y10, (c) F19, and (d) F20 residues.

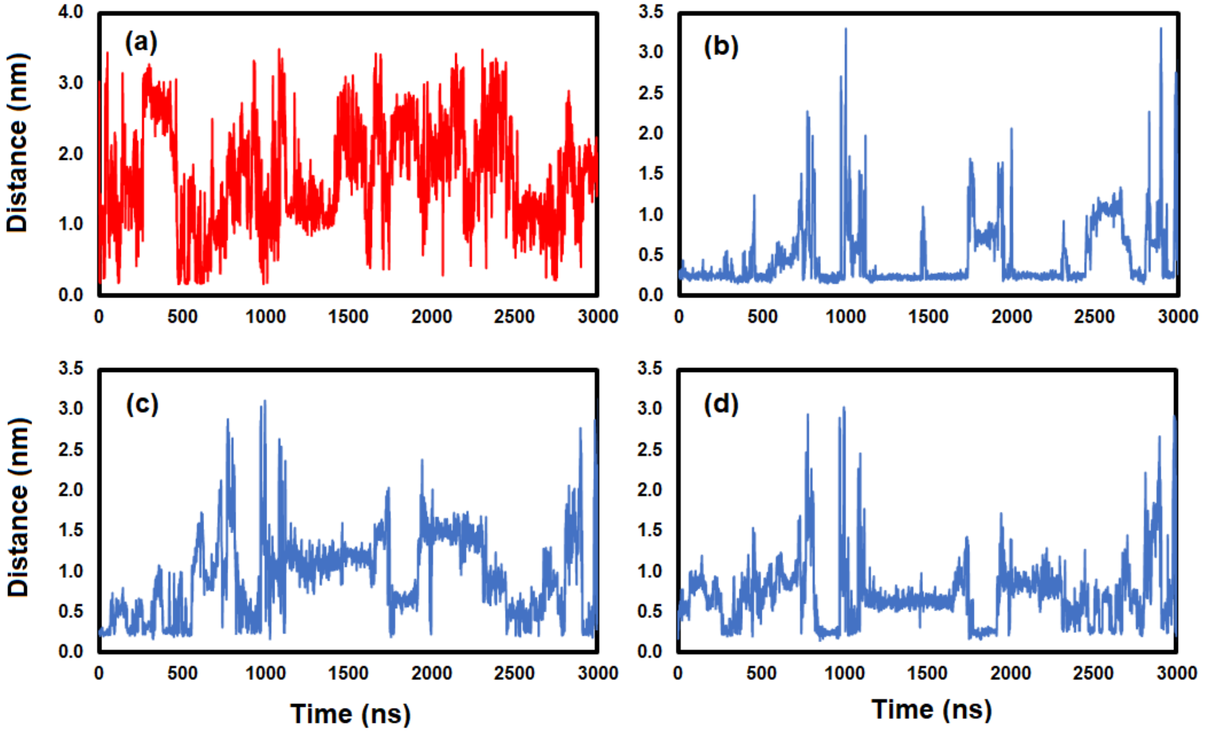

Figure S6. (a) Average Aβ_{42} end-to-end distance (D1–A42) and distances between **EC** and (b) Y10, (c) F19, and (d) F20 residues.

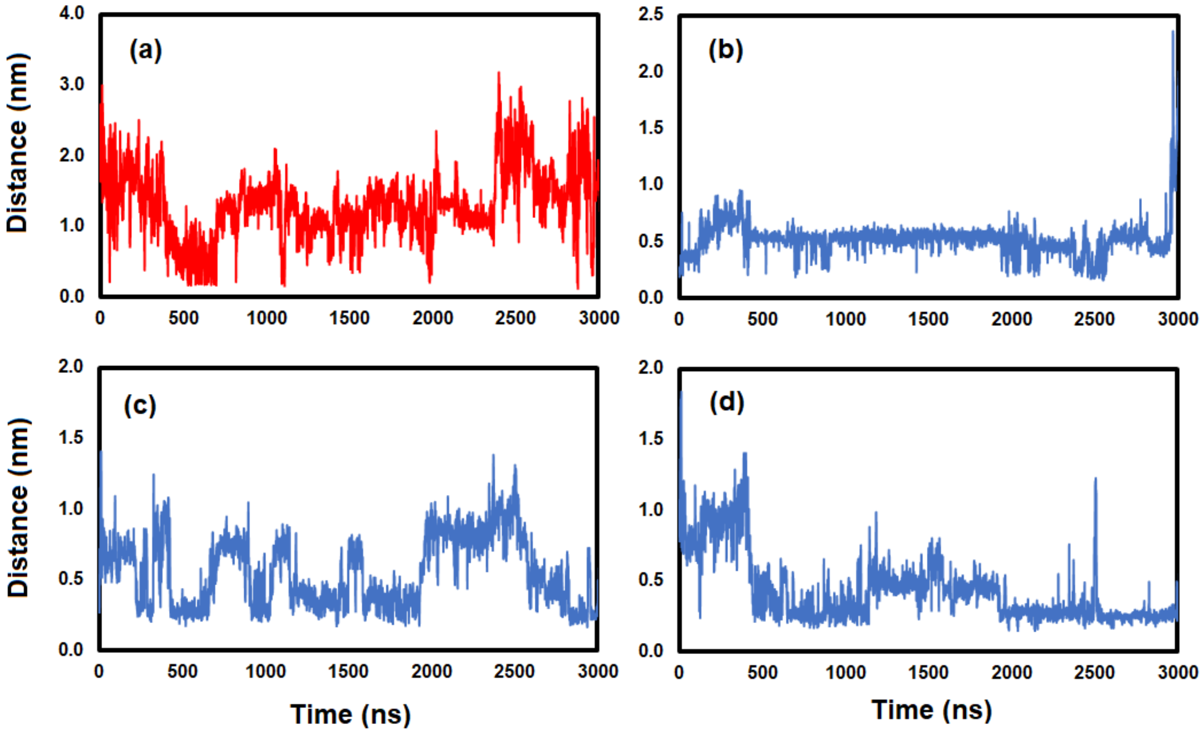

Figure S7. (a) Average Aβ_{42} end-to-end distance (D1–A42) and distances between ECG and (b) Y10, (c) F19, and (d) F20 residues.

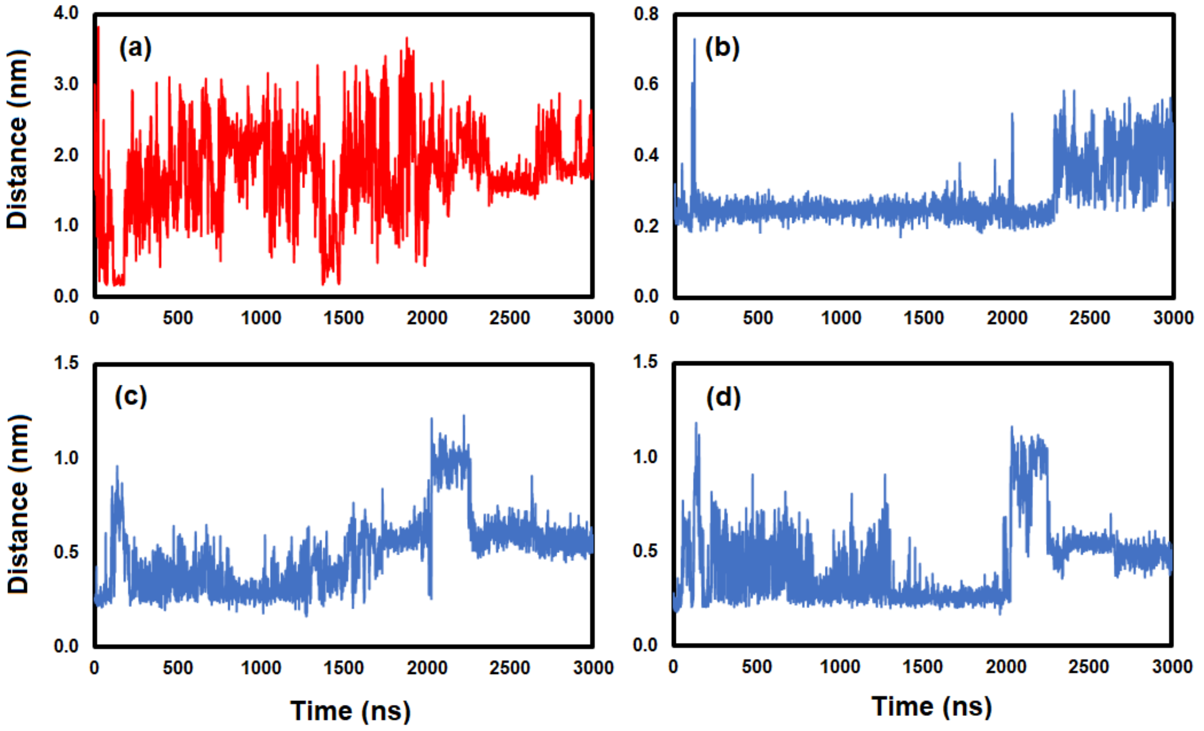

Figure S8. (a) Average Aβ_{42} end-to-end distance (D1–A42) and distances between EGC and (b) Y10, (c) F19, and (d) F20 residues.

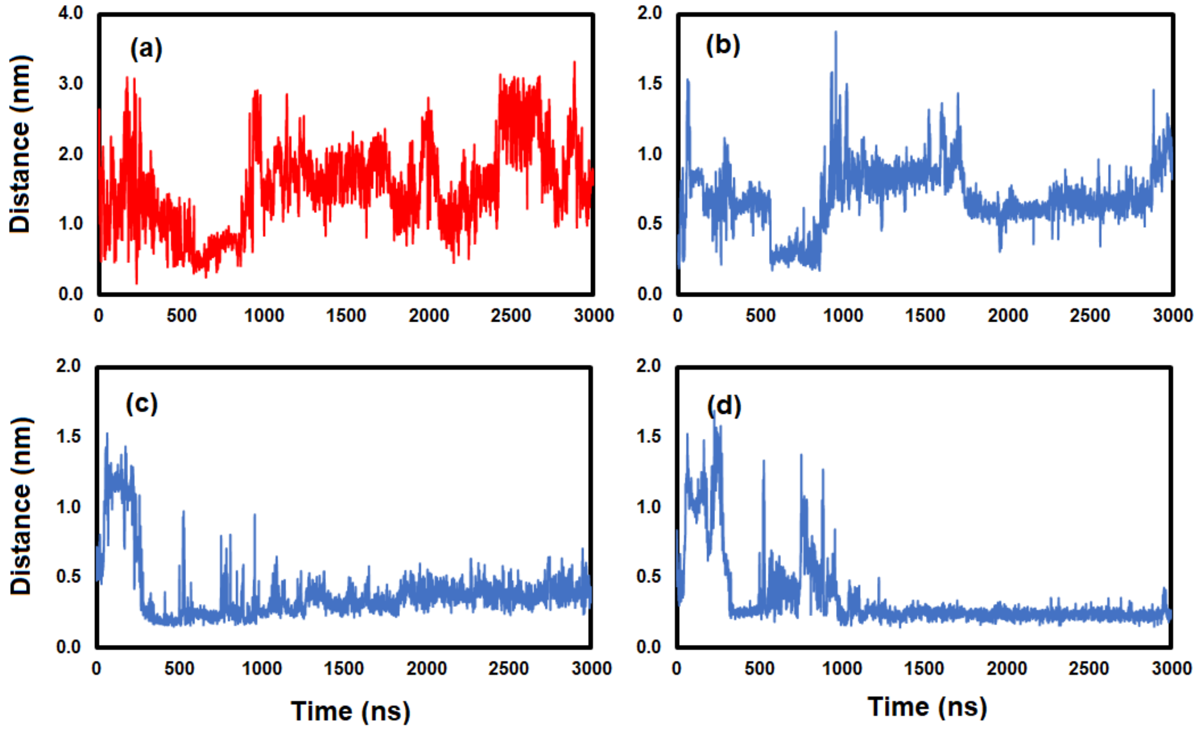

Figure S9. (a) Average Aβ_{42} end-to-end distance (D1–A42) and distances between EGCG and (b) Y10, (c) F19, and (d) F20 residues.

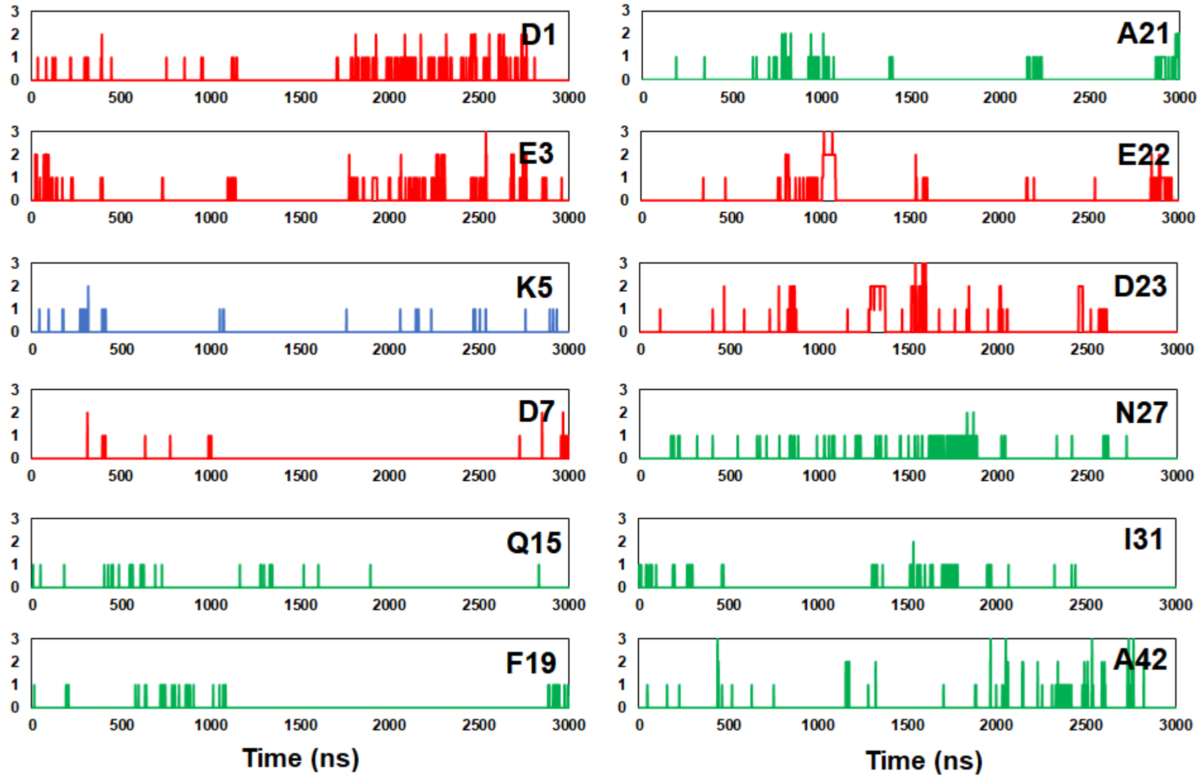

Figure S10. Hydrogen bonds between C and Aβ_{42} selected residues. Anionic, cationic, and neutral residues are shown in red, blue, and green, respectively.

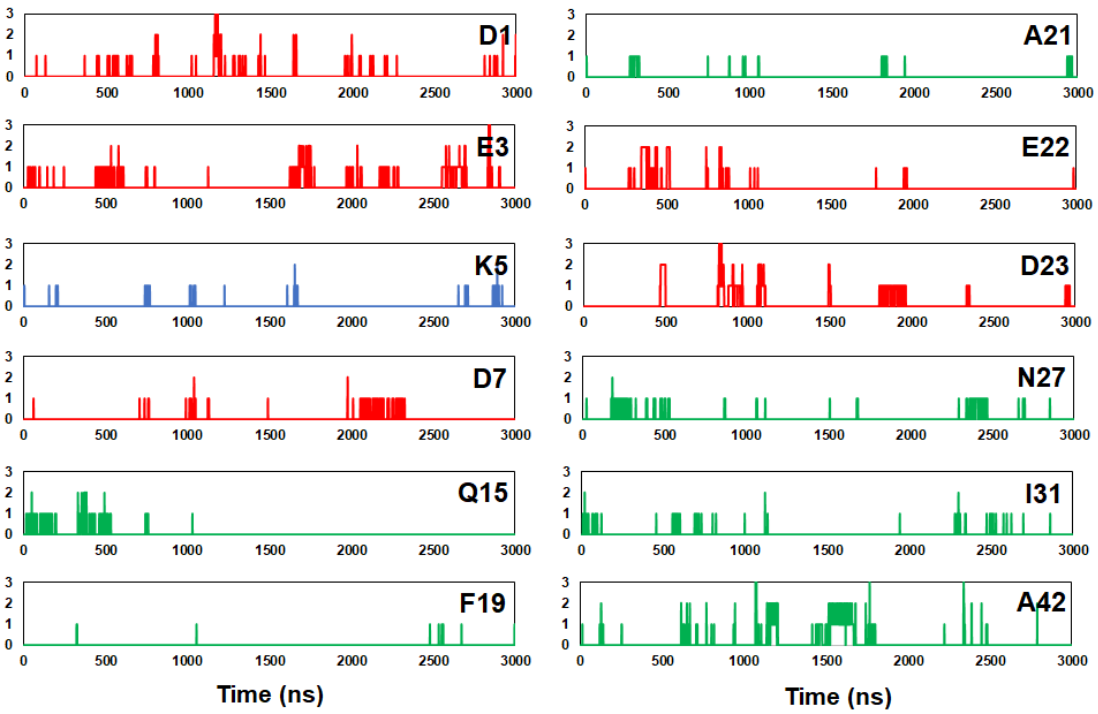

Figure S11. Hydrogen bonds between EC and Aβ_{42} selected residues. Anionic, cationic, and neutral residues are shown in red, blue, and green, respectively.

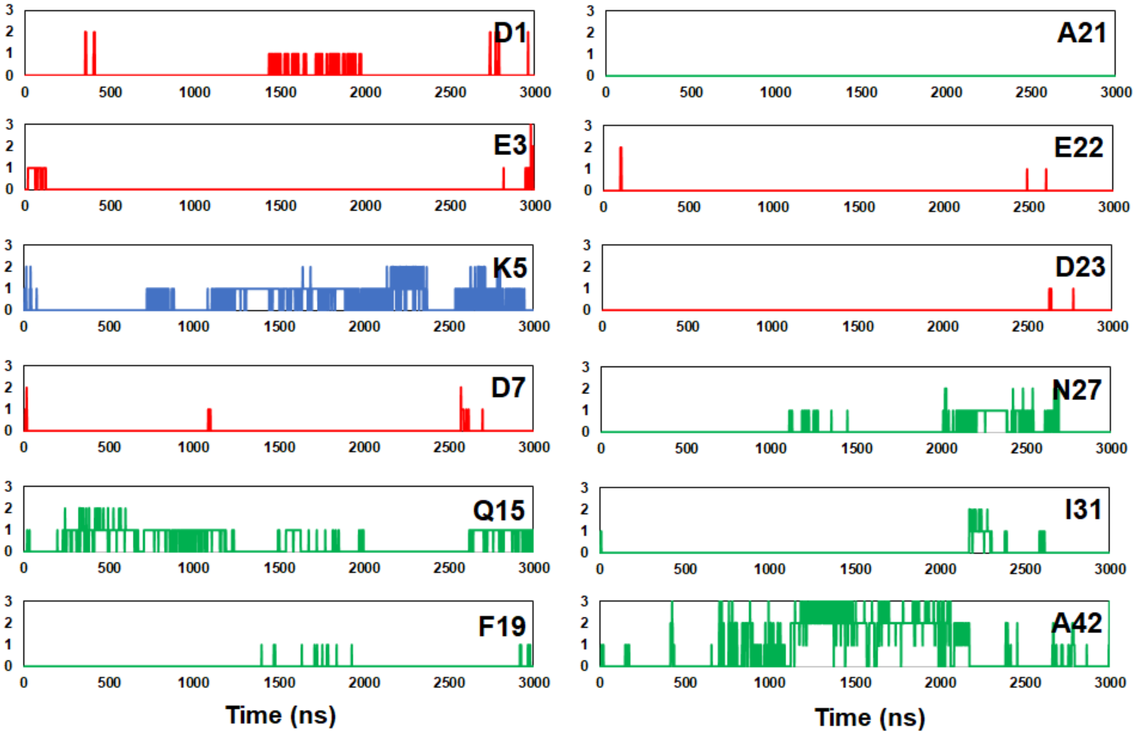

Figure S12. Hydrogen bonds between ECG and Aβ_{42} selected residues. Anionic, cationic, and neutral residues are shown in red, blue, and green, respectively.

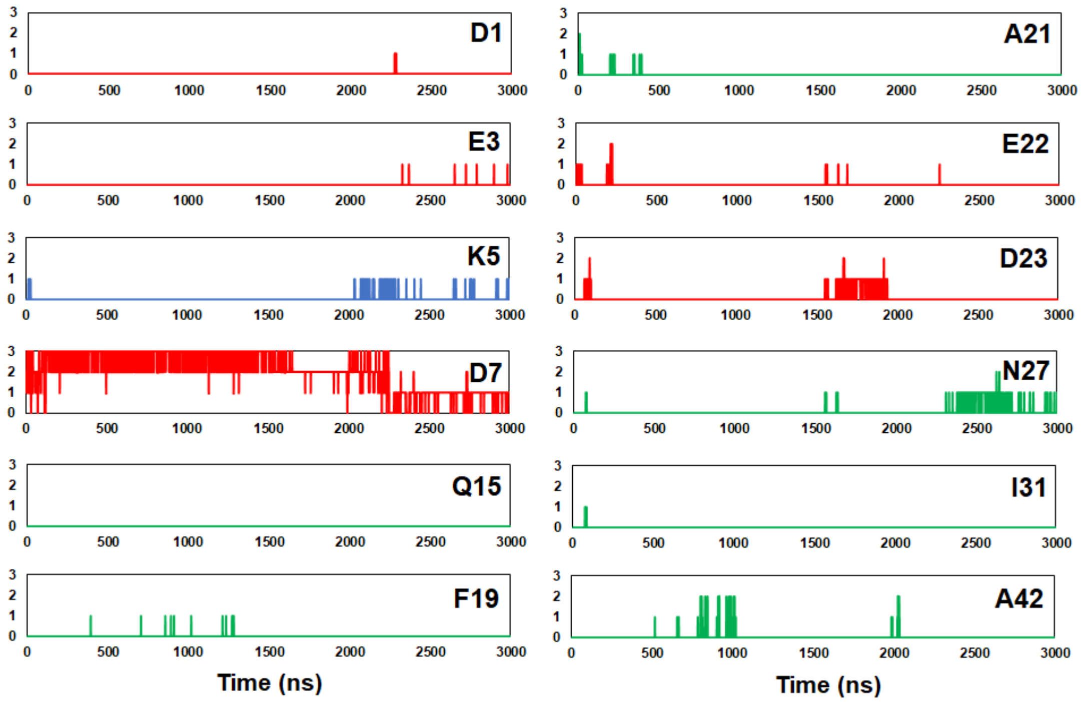

Figure S13. Hydrogen bonds between EGC and Aβ_{42} selected residues. Anionic, cationic, and neutral residues are shown in red, blue, and green, respectively.

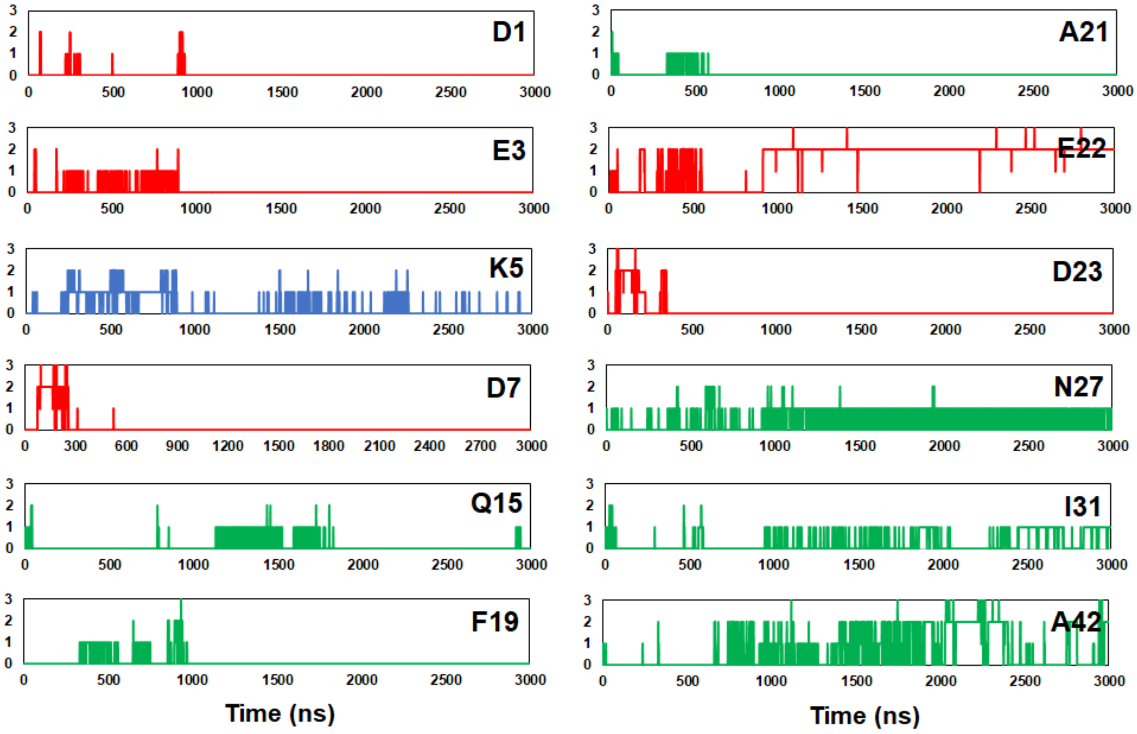

Figure S14. Hydrogen bonds between EGCG and Aβ_{42} selected residues. Anionic, cationic, and neutral residues are shown in red, blue, and green, respectively.

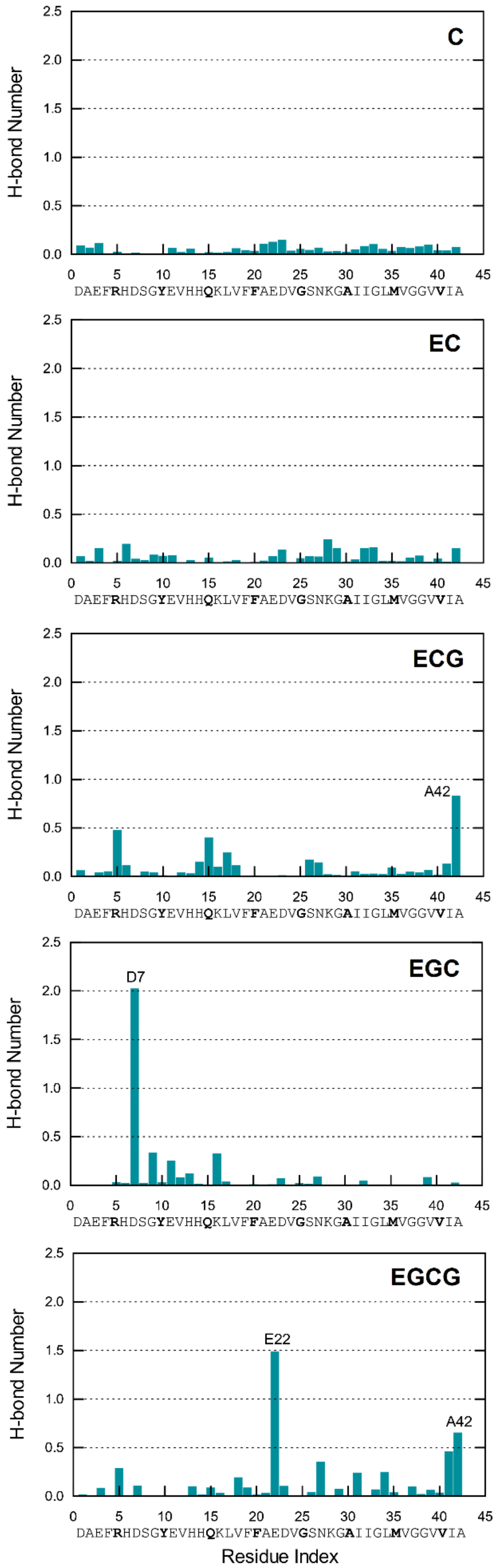

Figure S15. Average number of hydrogen bonds between Aβ_{42} amino acids and the ligands.

References

1. Eisenhaber, F.; Lijnzaad, P.; Argos, P.; Sander, C.; Scharf, M., The double cubic lattice method: Efficient approaches to numerical integration of surface area and volume and to dot surface contouring of molecular assemblies. *J. Comput. Chem*. **1995**, *16*, 273-284.
